# Type 1 secretion necessitates a tight interplay between all domains of the ABC transporter

**DOI:** 10.1038/s41598-024-59759-0

**Published:** 2024-04-18

**Authors:** Manuel T. Anlauf, Florestan L. Bilsing, Jens Reiners, Olivia Spitz, Eymen Hachani, Sander H. J. Smits, Lutz Schmitt

**Affiliations:** 1 Institute of Biochemistry, Heinrich Heine University Düsseldorf, Universitätsstraße 1, 40225 Düsseldorf, Germany; 2 Center for Structural Studies, Heinrich Heine University Düsseldorf, Universitätsstraße 1, 40225 Düsseldorf, Germany; 3 Present Address: INCONSULT, Duisburg, Germany

**Keywords:** Type I secretion system, ABC transporter, Hemolysin, Substrate recognition, Proteins, Membrane proteins

## Abstract

Type I secretion systems (T1SS) facilitate the secretion of substrates in one step across both membranes of Gram-negative bacteria. A prime example is the hemolysin T1SS which secretes the toxin HlyA. Secretion is energized by the ABC transporter HlyB, which forms a complex together with the membrane fusion protein HlyD and the outer membrane protein TolC. HlyB features three domains: an N-terminal C39 peptidase-like domain (CLD), a transmembrane domain (TMD) and a C-terminal nucleotide binding domain (NBD). Here, we created chimeric transporters by swapping one or more domains of HlyB with the respective domain(s) of RtxB, a HlyB homolog from *Kingella kingae*. We tested all chimeric transporters for their ability to secrete pro-HlyA when co-expressed with HlyD. The CLD proved to be most critical, as a substitution abolished secretion. Swapping only the TMD or NBD reduced the secretion efficiency, while a simultaneous exchange abolished secretion. These results indicate that the CLD is the most critical secretion determinant, while TMD and NBD might possess additional recognition or interaction sites. This mode of recognition represents a hierarchical and extreme unusual case of substrate recognition for ABC transporters and optimal secretion requires a tight interplay between all domains.

## Introduction

Secretion, the transport of a substrate from the cytosol across the membrane to the extracellular space or even to the cytosol of the host cell, is used by bacteria for several processes, e.g. acquirement of nutrients by scavenging proteins, cell-to-cell communication via quorum sensing or infection of host cells using toxins or other virulence factors. Today, at least nine secretion systems were identified in Gram-negative bacteria^1–3^. One of them, the Type I secretion system (T1SS, reviewed in Holland et al*.*^4^) is considered to be a prototype system. It has a rather simple architecture consisting of an ATP binding cassette (ABC) transporter and a membrane fusion protein (MFP) residing in the inner membrane as well as an outer membrane protein (OMP) present in the outer membrane. Together they form a continuous channel, allowing the transport of various substrates ranging from small peptides to huge S-layer proteins in an unfolded state in one step without the occurrence of a periplasmic intermediate^5^.

One of the best investigated T1SSs is the hemolysin system found in uropathogenic *Escherichia coli* (*E. coli*) strains^6,7^. Its MFP is hemolysin D (HlyD), its OMP is TolC, and as a member of the T1SS sub-family 2, its ABC transporter is hemolysin B (HlyB), which contains an N-terminal C39 peptidase-like domain (CLD)^8^. Although the CLD does not have peptidase activity, it was shown to modulate HlyB’s activity and to be essential for the secretion by interacting with the C-terminal part of the substrate hemolysin A (HlyA) in its unfolded state^8–11^. Sequence analysis revealed, that transporters involved in the secretion of *R*epeat in *T*o*X*in (RTX) proteins all carry such a CLD^8,10^. Additionally, it was shown that the nucleotide binding domain (NBD) of HlyB interacts with the secretion signal of HlyA^12^. A recent study identified two possible binding regions for HlyA in the NBD of HlyB as well as indications for a concerted binding of HlyA to both the CLD and NBD^13^. Only recently, the structure of the HlyB-HlyD complex was resolved by single particle cryo-EM^14^ and showed an unexpected stoichiometry composed of three HlyB dimers in complex with six HlyD.

HlyA is a 1,024 amino acids long pore-forming toxin and features several glycine-rich nonapeptide repeats (GG repeats), which are characteristic for the RTX domain and allow the binding of Ca^2+^ ions, which promotes the folding of the GG repeats into a β-roll^15^, but only in the extracellular space, since the bacterial intracellular Ca^2+^ concentration (approx. 300 nM) is several magnitudes lower than the K_D_ of HlyA (150 µM)^16,17^. HlyA is secreted via its C-terminal secretion signal, which is encoded within the last 48–60 amino acids and reaches the outside of the cell first^18^. Although research has focused on elucidation of common features for secretion signals of T1SS, universal conservation of the primary sequence does not exist^4^. This led to the suggestion, that the presence of a secondary structure element could be a prerequisite for a functional secretion signal. Multiple studies were performed to elucidate the presence of structural features in the signal sequence of HlyA and related proteins and the presence of an amphipathic helix was proven to be essential for the early steps of secretion^19–25^.

The hemolysin system was shown to exhibit a considerable promiscuity for the secreted substrate. For instance, cells expressing *hlyBD* were able to secrete LktA from *Pasteurella haemolytica*^26^, PaxA from *Pasteurella aerogenes*^27^, CyaA from *Bordetella pertussis*^28^, NodO from *Rhizobium leguminosarum*^29^, FrpA from *Neisseria meningitidis*^30^, HlylA from *Actinobacillus pleuropneumoniae*^31^, AqxA from *Actinobacillus equuli*^32^ and MbxA from *Moraxella bovis*^33^.

The above mentioned examples motivated us to further investigate the specificity of interactions between the substrate HlyA and the ABC transporter HlyB in this study. For this, we created different chimeras of HlyB (chHlyB) by swapping the CLD, transmembrane domain (TMD), NBD and/or combinations of these domains with the respective domain of a homologous T1SS ABC transporter from *Kingella kingae* (*K. kingae*), RtxB. *K. kingae* is a Gram-negative bacterium and an emerging pathogen infecting mainly young children, causing e.g. osteoarticular infections, septic arthritis and endocarditis^34–36^. Similar to *E. coli* and its hemolysin system, *K. kingae* is equipped with a T1SS and secretes the RTX protein RtxA^37^. Secretion experiments using our chimeric transporters suggest that all three domains of HlyB seem to contain identity determinants for recognition and secretion of HlyA, with the CLD being the most critical one, suggesting a hierarchy with the individual interactions and tight interplay of the domains.

## Results

### The Rtx system from *Kingella kingae*, a homolog to the hemolysin system

In our search of suitable, homologous candidates for the domain swapping of HlyB, we applied the protein Basic Local Alignment Search Tool (pBLAST) using the primary sequence of HlyB (UniProt-ID: Q1R2T6). We specifically searched for transporters, which lacked a cysteine residue in the first 100 amino acids, as this classifies the transporter as a group 2 T1SS ABC transporter with a CLD, just like HlyB^8,10^. After identification of such ABC transporters, the genome of the respective organism was scanned for the presence of a HlyD-like MFP using pBLAST with the sequence of HlyD (UniProt-ID: Q1R2T7) and for the presence of an RTX protein using pBLAST with the sequence of HlyA as a reference (UniProt-ID: P08715). Most organisms featured multiple proteins containing RTX motifs (noted in Supplementary Table S1). Organisms lacking an MFP or RTX protein were excluded from the list of interesting homologs. A total of 25 organisms identified were included for an alignment using Clustal Omega^38^ (Supplementary Table S1). The resulting phylogenetic tree could be subdivided into four groups (see Supplementary Fig. S1).

The ABC transporter RtxB from *K. kingae* has an identity of 71% when compared with HlyB. Taking a closer look on the domains of the ABC transporters, their TMDs and NBDs have the highest identity with 79% and 73% respectively, while the CLDs have an identity of only 48%. The toxin RtxA from *K. kingae* is composed of 956 amino acids and somewhat smaller than HlyA (1,024 amino acids), but still similar in size. The structural model for RtxA as predicted by AlphaFold2^39^ as well as secondary structure predictions reveal an amphipathic helix similar to HlyA (Supplementary Figs. S2, S3 and S4). However, the secretion signals (the last 100 amino acids) of HlyA and RtxA have a very low identity of 23%, with the sequence identity of the entire proteins being not much higher (42%). The MFPs HlyD and RtxD show a similar identity level as the toxins with 40%. We chose the T1SS from *K. kingae* and RtxB as the source for the domain swapping approach of HlyB based on the level of sequence identities (Supplementary Fig. S1). We also reasoned, that this combination was ideal to investigate the influence of the different ABC transporter domains on the substrate specificity without the risk of either no effect on secretion because of too high similarity, or inability to secrete with any domain combination because of too structurally different substrates.

**Figure 1:**
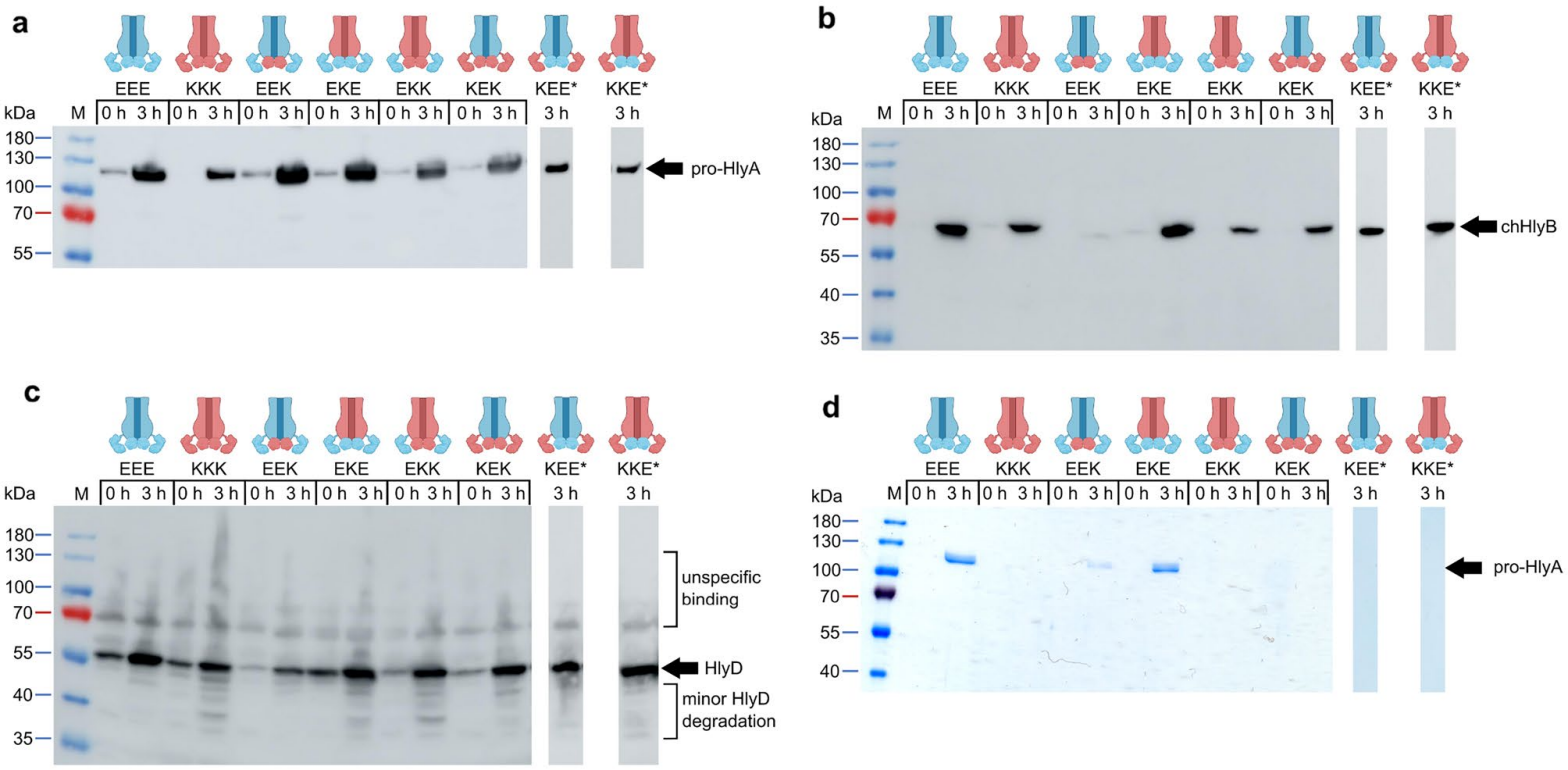
Secretion of pro-HlyA by chHlyB ABC transporters. Immunoblot analysis of whole *E. coli* cells expressing the indicated chHlyB show the presence of pro-HlyA (**a**), chHlyB (**b**) and HlyD (**c**). SDS-PAGE analysis (**d**) shows secreted pro-HlyA in the supernatant of *E. coli* cells expressing the different chHlyB, HlyD and pro-HlyA. The contrast of the SDS-PAGE in (d) was adjusted to improve visibility of secreted pro-HlyA in the case of HlyB-EEK. Schematic representation of the chHlyB variants are depicted above the respective chimera. Blue domains originate from HlyB (*E. coli*) and red domains from RtxB (*K. kingae*). Cell and supernatant samples were diluted to match the same OD_600_. Note that samples marked with an asterisk are not from the same Western blot membrane. Uncropped Western blots and SDS-PAGE gels are shown in Supplementary Fig. S5. Signals corresponding to unspecific binding and degradation are marked in case of the anti-HlyD western blot. M: Protein marker, the approximated molecular weight of the marker proteins is given on the left; x h: time after induction, when the samples were taken.

We therefore created chimeric HlyB (chHlyB) variants by swapping one, two or all three domains with the respective domain of RtxB. The 3-letter nomenclature used for the chimeras in this study is derived from the domains position; the first letter is designated for the N-terminal CLD, while the second and third letter represent the TMD and C-terminal NBD, respectively. The letter describes the origin of the domain, with ‘E’ being the domain from *E. coli* HlyB and ‘K’ being the one from *K. kingae* RtxB. For example, the ABC transporter ‘HlyB-EKE’ contains the N-terminal CLD and C-terminal NBD from HlyB, while the TMD is derived from RtxB. Likewise, HlyB-EEE is synonymous to HlyB and HlyB-KKK is synonymous to RtxB. A colored scheme for the transporter’s domain origin is provided above the respective SDS-PAGE and immunoblot analyses (Fig. 1).

### Secretion of pro-HlyA by HlyB-RtxB chimeras

We tested the ability of all chHlyB and RtxB (HlyB-KKK) to secrete the inactive pro-HlyA when co-expressed with HlyD in *E. coli* BL21(DE3) and compared it to HlyB. The inactive pro-HlyA lacks the acylation of two internal lysine residues, which is installed prior to secretion by the acyltransferase HlyC^40^. Immunoblot analysis targeting the secretion signal of pro-HlyA, the NBD of chHlyB or HlyD was performed to test for the expression of all proteins of the hemolysin T1SS. Only the presence of TolC was not tested, as it is an endogenous protein and constitutively expressed in *E. coli*^41–44^. Irrespective of the chHlyB variant, cells were able to express pro-HlyA (Fig. 1a) and HlyD (Fig. 1c). Cells expressing RtxB showed a slightly lower expression level of pro-HlyA. The expression level of HlyB-EEK was visibly reduced, and the co-expression of HlyD in the same cells was lower when compared to other chimeric transporters (Fig. 1b). Overall, the signal intensity of transporters with the RtxB-NBD appeared reduced in comparison to transporters with the HlyB-NBD. All other cells were expressing HlyB, RtxB or chHlyB in comparable amounts. Nevertheless, not all HlyB variants were able to secrete pro-HlyA (Fig. 1d). All transporter variants carrying the CLD from RtxB failed to secrete pro-HlyA. Only HlyB and the chimeras HlyB-EEK and HlyB-EKE showed a signal for pro-HlyA in the supernatant. For HlyB-EEK, the amount of secreted protein was often difficult to visualize using Coomassie staining (Fig. 1d). Therefore, detection via immunoblot analysis was additionally carried out later on. Interestingly, exchanging either only the TMD or only the NBD of HlyB to the respective version of RtxB allowed the secretion of pro-HlyA. On the other hand, swapping both domains at the same time and only keeping the CLD of HlyB (EKK) still rendered the transporter unable to secrete pro-HlyA.

### Quantification of secretion efficiencies of HlyB-EKE and HlyB-EEK

**Figure 2:**
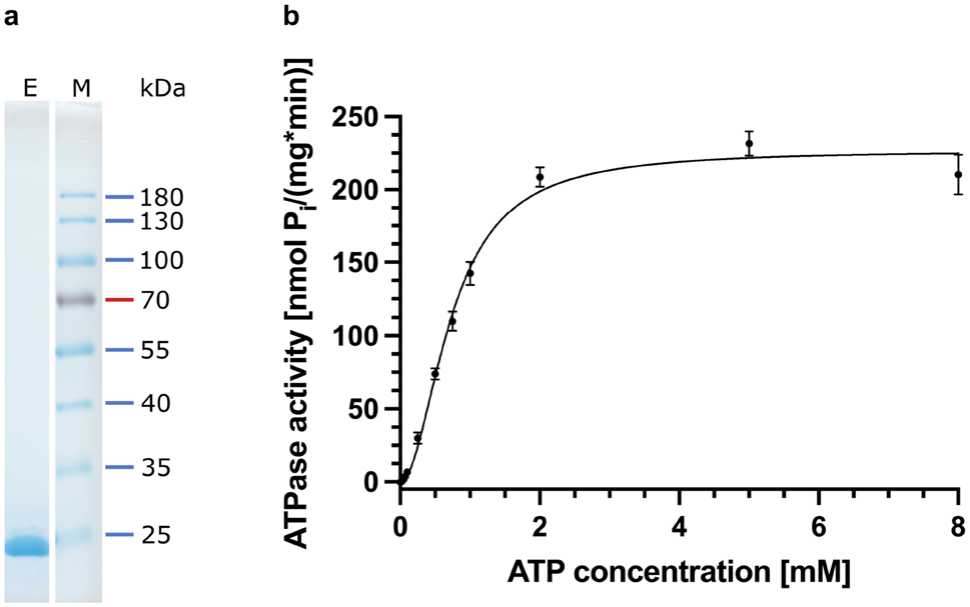
Analysis of purified RtxB-NBD. (**a**) SDS-PAGE analysis of purified RtxB-NBD. Shown is the elution fraction (E) after Size Exclusion Chromatography with Coomassie staining. Chromatograms of the purification and an uncropped SDS-PAGE analysis of additional samples are shown in Supplementary Fig. S7. RtxB-NBD-NHis6 has a size of 27,676 Da. M: Protein marker, the approximated molecular weight of the marker proteins is given on the right. (**b**) ATPase activity of RtxB-NBD in dependency of varying ATP concentrations. The data was analyzed using the Hill equation. Shown are mean values and standard deviations as error bars of three independent experiments.

We focused on the transporters, which were competent in transporting pro-HlyA and aimed to quantify the secretion efficiencies for HlyB-EKE and HlyB-EEK in comparison to HlyB (HlyB-EEE). Since we noticed a reduced band intensity of transporters carrying the RtxB-NBD we intended to determine if the HlyB-NBD antibody recognized the NBD of RtxB with a reduced efficiency. Note that the anti-HlyB antibody was raised against the NBD of HlyB. For this, we overexpressed and purified the soluble RtxB-NBD with an N-terminal 6xHis-tag (theoretical molecular weight 27,676 Da) to homogeneity as shown by SDS-PAGE and Small-angle X-ray scattering (SAXS) analysis (Supplementary Table S2, Fig. 2a and Supplementary Figs. S7 and S8). We subjected pure HlyB-NBD and RtxB-NBD to an SDS-PAGE and quantified the intensity of the NBD signals with the HlyB-NBD antibody (Supplementary Fig. S9). Even though equal amounts of protein were used in both cases, the signal intensity ratio between the RtxB-NBD and the HlyB-NBD was 0.36 ± 0.10 when using the HlyB-NBD antibody. Accordingly, the quantified secretion efficiency of HlyB-EEK was corrected using the aforementioned factor to account for the decreased recognition of the NBD from *K. kingae* by the used antibody.

**Figure 3:**
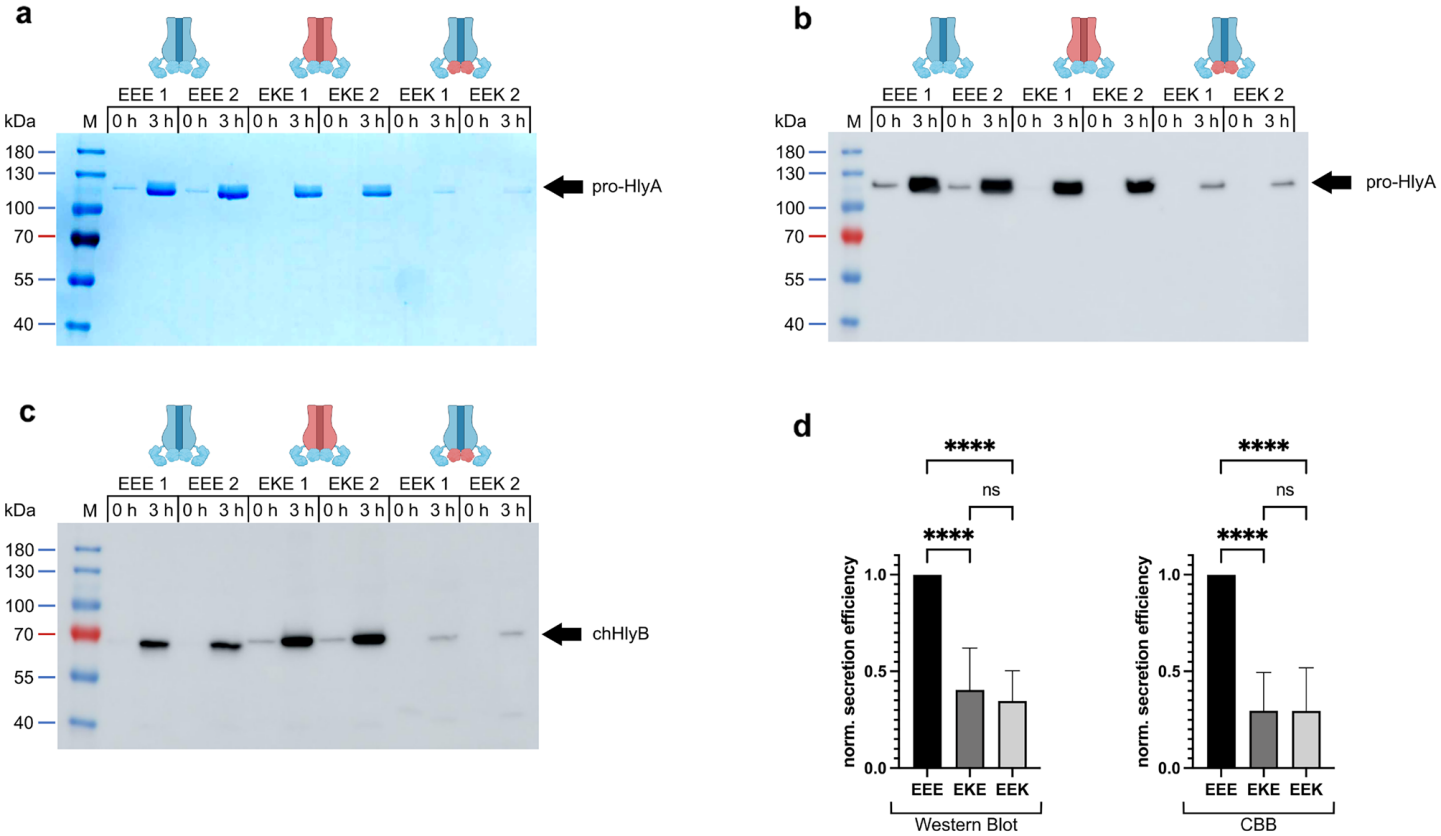
Quantification of chHlyB expression and pro-HlyA secretion. SDS-PAGE (**a**) and immunoblot analysis (**b**) of pro-HlyA from supernatants of *E. coli* cells expressing the indicated chHlyB and HlyD in two replicates; the used antibody targeted the secretion signal of pro-HlyA. Immunoblot (**c**) of whole *E. coli* cells show the amount of expressed chHlyB in two replicates; the used antibody targeted the NBD of chHlyB. The samples were diluted to match the same OD_600_. The contrast of the SDS-PAGE in **(**a**)** was adjusted to improve visibility of secreted pro-HlyA in the case of HlyB-EEK. Schematic representation of the chHlyB variants are depicted above the respective chimera. Blue domains originate from HlyB (*E. coli*) and red domains from RtxB (*K. kingae*). The quantification revealed reduced secretion efficiencies for both HlyB-EKE (dark grey) and HlyB-EEK (light grey) by a factor of ~ 3 when compared to HlyB-EEE (black)**.** Uncropped Western blots and SDS-PAGE gels are shown in Supplementary Fig. S6. (**d**) The left panel shows the calculations using signals from Western blots for quantifications of pro-HlyA, while the right panel shows quantifications using Coomassie stained gels (CBB). Shown are the mean values with standard deviations as error bars (n ≥ 16, biological replicates). Statistical analysis was performed using a one-way ANOVA test (****p < 0.0001, ns: p = 0.5138 for EKE vs. EEK (Western Blot) and p = 0.9995 for EKE vs. EEK (CBB)). M: Protein marker, the approximated molecular weight of the marker proteins is given on the left; x h: time after induction, when the samples were taken.

We repeated the secretion experiments with HlyB, HlyB-EKE and HlyB-EEK to determine relative secretion efficiencies. For this, we quantified the amount of pro-HlyA secreted into the supernatant (Fig. 3a,b) as well as the amount of the HlyB variants in whole cells (Fig. 3c). Normalizing the protein amounts of the chimeric ABC transporters as well as the secreted pro-HlyA to the ones of HlyB-EEE, and dividing the amount of secreted pro-HlyA by the amount of chHlyB in the cells, allowed the determination of relative secretion efficiencies (Fig. 3d). With this approach, the secretion efficiency of HlyB-EEE was set to 1.0. For both, HlyB-EKE and HlyB-EEK, secretion was reduced by a factor of approximately three. The calculations were performed with secreted pro-HlyA quantified by immunoblot analysis (HlyB-EKE: 0.41 ± 0.22; HlyB-EEK: 0.35 ± 0.16) and from Coomassie stained gels (HlyB-EKE: 0.30 ± 0.20; HlyB-EEK: 0.30 ± 0.22). The reason for the reduced secretion of HlyB-EKE and -EEK seem to be different and synergistic, as the secretion of pro-HlyA by the chimera HlyB-EKK, in which both substitutions of the TMD and NBD were combined, was completely abolished (Fig. 1).

### ATPase activity of RtxB-NBD

Next, we examined, if the reduced secretion of pro-HlyA by HlyB-EEK could be ascribed to a decreased ATP hydrolysis rate. For this, the ATPase activity of RtxB-NBD was determined colorimetrically (Fig. 2b) using Eq. (1). The kinetic parameters are summarized in Table 1 and compared to the HlyB-NBD^13^. For RtxB-NBD, the maximum velocity (v_max_) was calculated to be 227.0 ± 2.9 nmol P_i_ mg^−1^ min^−1^ and therefore comparable to the maximum velocity of 253.8 ± 7.6 nmol P_i_ mg^−1^ min^−1^ for HlyB-NBD^13^. Both the hill coefficient (h = 1.96 ± 0.10) and the ATP concentration providing half the maximum velocity (K_0.5_ = 0.74 ± 0.02 mM) of RtxB-NBD were slightly increased when compared to HlyB-NBD (h = 1.66 ± 0.154; K_0.5_ = 0.49 ± 0.026 mM)^13^. Still, these differences in kinetic parameters are only minor and do not explain the major reduction in HlyA secretion efficiency of HlyB-EEK.

**Table 1:** Kinetic parameters determined for RtxB-NBD.

Kinetic parameter	RtxB-NBD	HlyB-NBD^13^
v_max_ [nmol P_i_ mg^−1^ min^−1^]	227.0 ± 2.9 (s.d.)	253.8 ± 7.6
K_0.5_ [mM]	0.74 ± 0.02 (s.d.)	0.49 ± 0.03
h	1.96 ± 0.10 (s.d.)	1.66 ± 0.15

The maximum enzyme velocity (v_max_), the substrate concentration at half maximal enzyme velocity (K_0.5_) as well as the Hill coefficient (h) are displayed for RtxB-NBD and compared to the parameters of HlyB-NBD^13^.

## Discussion

The hemolysin system is often referred to as the prototypic T1SS. Its ABC transporter HlyB is enabling the active transport of the toxin HlyA by binding and hydrolyzing ATP through its NBD. The TMD is anchoring the transporter in the inner membrane and forming the translocation channel, as shown by crosslinking studies and the recently published structure of the HlyB-HlyD complex^11,14^, while the CLD is important for substrate recognition and modulation of hydrolytic activity^10,11^. Recent studies also showed that the NBD of HlyB is also interacting with its substrate, thus not only energizing the secretion process^12,13^. The secretion signal sequence with a putative amphipathic helix within HlyA is of utmost importance for this interaction^25^. HlyB has shown to exhibit at least some promiscuity in recognizing and secreting substrates^26–33^. Here, we have used chimeric transporters of the T1SS from *E. coli* and *K. kingae* to shed light onto specificity determinants within the domains of the transporter HlyB for the recognition and secretion of its substrate HlyA.

Our secretion assays highlight that the CLD is the most important specificity determinant for the secretion of HlyA (Fig. 1). No pro-HlyA was detectable in supernatant samples of chHlyB, in which the CLD was exchanged to the one of RtxB, a HlyB homolog from *K. kingae*. Former investigations showed that the deletion of the CLD from HlyB renders the transporter unable to secrete^10^. The same study showed a specific interaction of the CLD with the unfolded RTX domain of HlyA. Even though RtxA and HlyA share the same number of GG repeats in their RTX domains and the fold of RTX domains is conserved, the premise for the CLD-substrate interaction seems to be the exact amino acid sequence. This assumption is supported by the facts, that HlyA is present in an unfolded state in the cytoplasm and that the binding of the HlyB-CLD was only observed with unfolded HlyA^10^, indicating that the RTX fold itself is not recognized. Furthermore, the CLD possesses the lowest identity of all three domains when HlyB is compared to RtxB. Therefore, it is likely that the CLD evolved to specifically recognize its associated substrate and the simple presence of a CLD, even a homologous one, is not sufficient for recognition and secretion of the endogenous substrate.

The secretion experiments further revealed, that HlyB tolerated the non-simultaneous swapping of either the TMD (HlyB-EKE) or NBD (HlyB-EEK) to the respective one from RtxB (Figs. 1 and 3). The single-substitutions were accompanied by a decrease of HlyA secretion with a similar factor when normalized to the expression level of the chimeric ABC transporter. Both domain swaps are likely to affect different aspects or steps of the HlyA secretion process, since combination of both substitutions into HlyB-EKK resulted in a transporter completely deficient in HlyA secretion. A lesser activity of the RtxB-NBD as a cause for the reduced secretion efficiency with HlyB-EEK is unlikely, since we purified the RtxB-NBD to homogeneity (Supplementary Table S2, Fig. 2a, Supplementary Figs. S7 and S8) and the determined kinetic parameters show an activity similar to HlyB-NBD (Table 1). Admittedly, the full-length RtxB might behave differently from the isolated domain. A recent study identified binding pockets for HlyA within the NBD of HlyB and a simultaneous binding of HlyA to the CLD and NBD of HlyB^13^. In our aim to address the question, why HlyB-KEE fails to recognize and secrete pro-HlyA, we could identify D81 in the RtxB-CLD as an interesting residue, since the corresponding position in HlyB is R82. The change of an aspartate to an arginine leads to a changed surface potential of the CLD in one area (Supplementary Fig. S10). We tested if a respective point mutation in HlyB-KEE, namely HlyB-KEE-D81R, could rescue the ability of the transporter to secrete pro-HlyA. However, this was not the case (Supplementary Fig. S11a–e), as no pro-HlyA was secreted by HlyB-KEE-D81R. Surprisingly, the overall expression of pro-HlyA in cells carrying this transporter was decreased (Supplementary Fig. S11a) and comparable to leaky pro-HlyA expression at the time of induction (0 h) observed for cells expressing HlyB-EEE or HlyB-KEE, while the expression level of the other T1SS components was similar (Supplementary Fig. S11b,c). An impairment of secretion or pro-HlyA detection in supernatants due to the decreased pro-HlyA expression in the case of HlyB-KEE-D81R is unlikely, since even trace amounts of pro-HlyA secreted by HlyB-EEE during leaky expression were detectable in the immunoblot (0 h, Supplementary Fig. S11d). Therefore, other or additional residues/surface patches must be involved.

**Figure 4:**
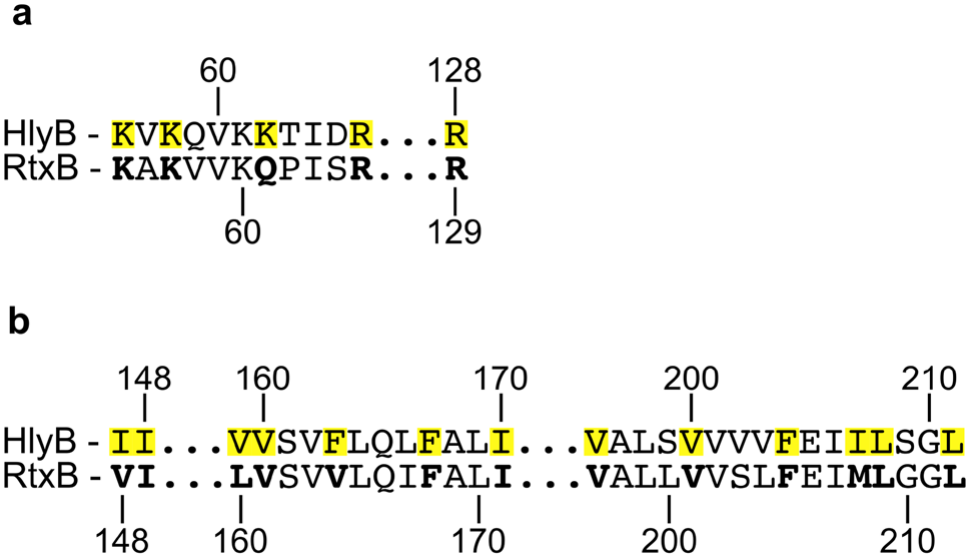
Comparative view on the CLD and TMD of HlyB and RtxB. Basic residues in the CLD (**a**) and hydrophobic residues in the TMD (**b**) of HlyB, which are involved in interactions with HlyD, are marked in yellow^14^. The respective residues in RtxB are highlighted as bold letters. A pairwise sequence alignment was performed using the GGSEARCH2SEQ tool^38^.

The reduced secretion efficiency of HlyB-EKE could be ascribed to additional critical interaction point(s) for HlyA within the TMD of HlyB, which are missing in the RtxB-TMD. Crosslinking studies already confirmed HlyA to travel through the channel formed by the HlyB-TMD^11,14^. So far it is not known, if the identified positions are critical interaction points or merely residues to which HlyA is in close proximity during the passage of the TMD. The classical view of ABC transporters and their specificity suggests a substrate binding pocket within the TMD in the inward facing conformation^45^. It is unlikely that the binding of HlyD to the chimeric transporter or that the multimeric complex assembly is affected: The identity of both MFPs HlyD and RtxD might be rather low with 40%. However, the cryo-EM structure of the HlyB-HlyD complex demonstrated, that the main interactions stabilizing the components in the inner membrane are provided by a cluster of acidic residues in HlyD and five basic residues in the CLD of HlyB^14^. Four of these five basic residues (K56, K58, R66 and R128) are also conserved in the CLD of RtxB and only one lysine, K62, is a glutamine in RtxB (Fig. 4a and b). Zhao et al. could show that complex assembly is rather stable as single substitutions of those basic residues did not affect the secretion of HlyA and only combinations of at least two substitutions resulted in a reduced or abolished secretion^14^. Additional contacts are made between the N-terminal region of HlyD and the transmembrane helices 1 and 2 of HlyB. Comparing the sequences of RtxB and HlyB, all of these residues are either identical or substituted by another hydrophobic amino acid^14^.

Pimenta et al*.* showed that the stability of HlyD is diminishing in the absence of HlyB (or TolC), most likely because HlyD is unstable in the absence of the other proteins of the secretion complex^46^. We noticed bands with smaller molecular weight for some of the chimeric transporters, but not to an extent as shown by Pimenta et al*.* or when we expressed a Strep-tagged variant of HlyD without HlyB present (Supplementary Fig. S12). Furthermore, the amount of degradation did not coincide with the observed secretion behavior. For example, HlyD showed less degradation in cells expressing HlyB-KEK, -KEE and -KKE (with which secretion of pro-HlyA was abolished) when compared to cells expressing HlyB-EKE or -EKK (the former was able to secrete pro-HlyA, while the latter was not) (Fig. 1c). Pimenta et al*.* noted, that HlyD is inherently prone to degradation, even in the presence of all transport components^46^, and we detected degradation bands of HlyD in cells expressing the wildtype transporter HlyB as well (Fig. 1c). HlyD on its own is only stably expressed when N-terminally truncated^47^. We therefore conclude, that the assembly of the secretion system is not affected and that the observed secretion behavior of the chimeric transporters is due to affected interaction with the substrate.

**Figure 5:**
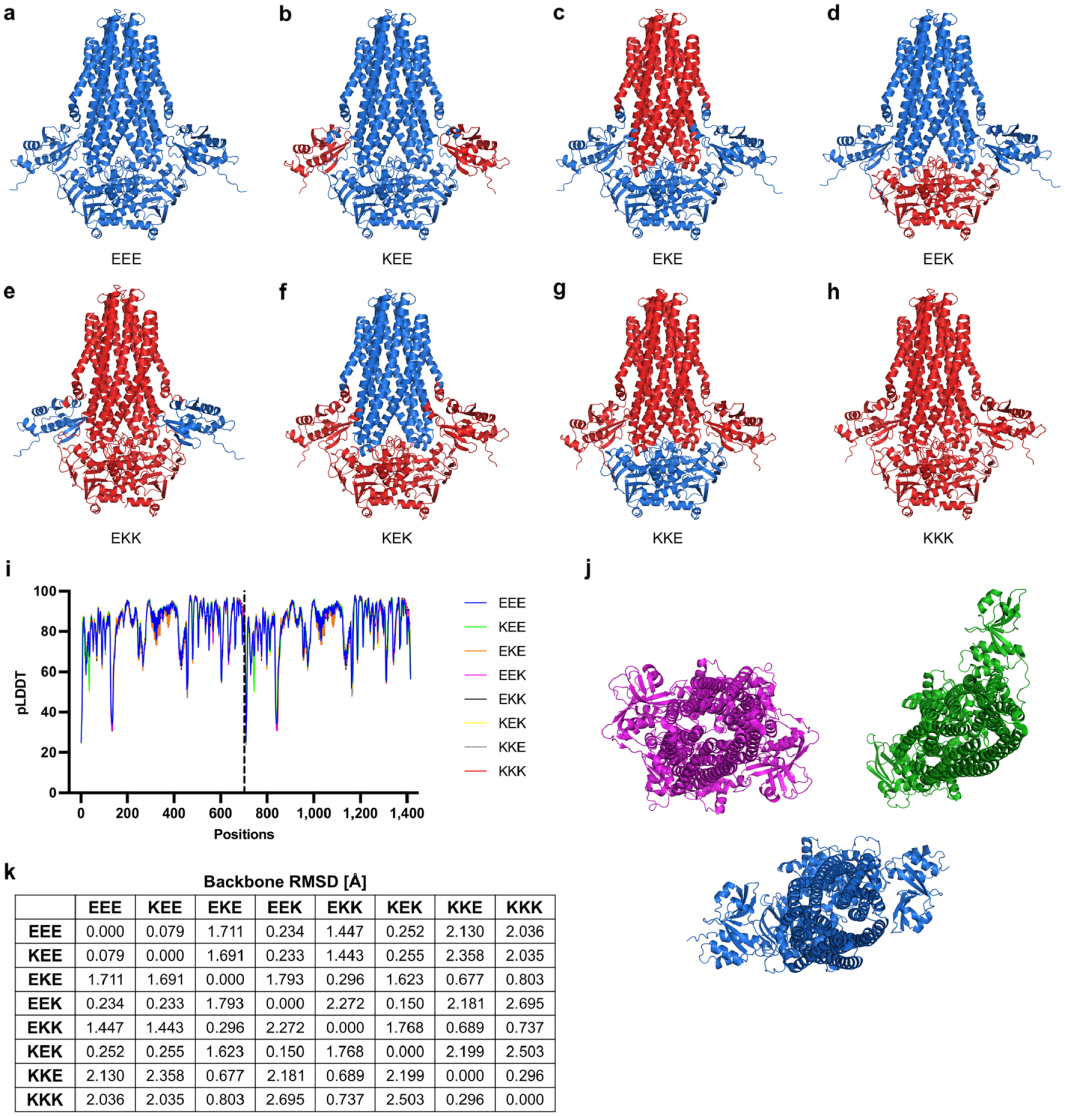
AlphaFold2 models of chimeric transporters used in this study and their model confidence. Depicted are the structural models of HlyB-EEE (**a**), HlyB-KEE (**b**), HlyB-EKE (**c**), HlyB-EEK (**d**), HlyB-EKK (**e**), HlyB-KEK (**f**), HlyB-KKE (**g**) and HlyB-KKK (**h**). The color code is the same as in the main text. (**i**): Graphical visualization of the pLDDT score for all Cα positions of the dimeric transporters used in this study. The dotted line separates the two monomers. (**j**): Orientation of the CLD of HlyB from the cryo EM structure (green, PDB: 7SGR), of the CLD of HlyB-EEE from the AlphaFold2 model (blue) and of the PEP domain of PCAT1 from the cryo EM structure (magenta, PDB: 6V9Z). (**k**): Root mean square deviation (RMSD) in Å between backbones of chimeric transporters used in this study.

A misfolded or unfolded CLD is unlikely to be the reason for the lack of secretion in respective chimeras. The CLD is located in the N-terminal part of the protein and folds first when emerging from the ribosome, irrespective of the identity of the following TMD and NBD. Furthermore, C39 peptidase and C39 peptidase-like domains are folding in the absence of the TMD and NBD of the transporter e.g. HlyB-CLD^10^ (PDB: 3ZUA), PCAT1-PEP^48^ (PDB: 7N87) or the peptidase domain of ComA^49^ (PDB: 3K8U). An assessment of correct CLD folding is difficult, since the CLD exhibits no peptidase activity and shows a high flexibility in the cryo EM structure^14^. We addressed the question of properly folded CLD using AlphaFold2. We created models for all chimeric transporters (Fig. 5), as well as RtxB and HlyB, and used the values of the predicted local-distance difference test (pLDDT) as a measure for likeliness of folding (see Fig. 5a–i).

High pLDDT scores have a high confidence score for the predicted fold, while low scores (below 50) may indicate unstructured regions. We included an AlphaFold2 model of HlyB-EEE, even though a cryo EM structure is available, to compare the values of chimeric transporters and RtxB with values of a transporter, which is known to fold properly. The AlphaFold2 models of all (chimeric) HlyB transporters used in this study show highly similar structures with backbone root mean square deviations (RMSDs) between the dimeric transporter models below 3 Å (Fig. 5k). Simultaneously, pLDDT values indicate no decreased likeliness of overall CLD folding in any (chimeric) transporter (Fig. 5i). The lowest pLDDT score of ~ 35 was calculated for the loop region connecting the CLD and TMD. The low score is likely originating from the high diversity in orientation of this loop between different ABC transporters (Fig. 5j). This loop (approximately residues 130–140) orients the CLD to the side in case of the cryo EM structure of HlyB (Fig. 5j, green) while the PEP domain of the cryo EM structure of substrate-bound PCAT1 is flipped upside down in comparison to the HlyB-CLD and oriented in front of the TMD interface (Fig. 5j, magenta). This loop adopts an intermediate form in the AlphaFold2 model of HlyB-EEE, as the CLD is oriented in front of the TMD interface, similarly to PCAT1, but not flipped (Fig. 5j, blue).

This study used chimeric transporters of HlyB, where one or more complete domains were swapped to the RtxB domains. With this, a detailed mapping of interaction sites within HlyB to HlyA, especially within the TMD and NBD, is not possible yet. This may be achieved by substituting only specific amino acids or patches of HlyB instead. The exchange of whole domains bears the risk of a non-native domain organization, which could influence functionality of the transporter, especially for fine-tuned interactions of the NBD and TMD via for example their coupling helix. Should this interaction be affected, e.g. for chimeras HlyB-EKE or HlyB-EEK, then the use of both RtxB domains (TMD and NBD) should grant full functionality. This is not the case, as we observed the opposite and secretion with HlyB-EKK was abolished (Fig. 1). Our domain swapping strategy demonstrated that HlyB shows a hierarchical substrate recognition involving all three domains with the CLD, although lacking peptidase activity, being the most important factor for the secretion of the substrate HlyA. Nevertheless, we cannot exclude that interactions of the CLD with the TMD or NBD or both or that differences in the interaction between the domains modulate this hierarchy. This is an unusual mode of action, as the classical ABC transporter binding pocket is located within the TMD^50,51^. A similar multi-domain interaction was reported for the Has system from *Serratia marcescens*^52^. However, this T1SS belongs to another subgroup and works fundamentally different compared to the HlyA system^8^: (i) the ABC transporter HasD has the classical architecture with no additional N-terminal domain, (ii) the substrate HasA is considerably smaller (~ 19 kDa), which features no RTX motifs, (iii) HasA is not secreted with its C-terminus first, but with its N-terminus first, (iv) the secretion signal is not involved in the assembly of the secretion complex, but its disassembly, (v) secretion of HasA is dependent on the general chaperone SecB, while no chaperones are known for the HlyA T1SS^8,52,53^. The identity of the TMD also plays a role in the recognition and/or secretion of HlyA by HlyB, but to a lesser extent when compared to ABC transporters, which translocate smaller molecules, like the multidrug resistance transporters MRP1 and PDR5^54,55^ or the lipopolysaccharide transporter LptB_2_FGC^56^. Equally important for the secretion is the identity of the NBD. Thus, one can speculate that the substantially larger substrate HlyA (110 kDa) might have necessitated multiple recognition sites in the transporter for a controlled secretion process.

## Material and methods

### Bacterial strains and plasmids

*Escherichia coli* DH5α cells were used for cloning, *E. coli* BL21(DE3) cells were used for overexpression, purification and secretion experiments. Plasmids and oligonucleotides used are listed in Supplementary Tables S3 and S4 respectively. The gene encoding for *rtxB* was isolated from the genome of *K. kingae* ATCC 23330 (DSM 7536) and ordered from the German Collection of Microorganisms and Cell Cultures GmbH (DSMZ).

### Construction of plasmids

The pK184-RtxB-HlyD plasmid as well as plasmids containing chimeric transporters were generated using Gibson assembly^57^. For this, pK184-HlyBD was amplified either without the domains of *hlyB* to be exchanged or without the complete gene *hlyB*. Additionally, the gene or domains of RtxB (UniProt-ID: F5S9L7) to be inserted were amplified from the genome of *K. kingae* (DSM 7536) with overlaps to the aforementioned pK184 plasmid. The purification plasmid pPSG122-RtxB-NBD-NHis6 was constructed similarly by amplifying the backbone of pPSG122-HlyB-NBD-NHis6^58^ without the *hlyB* NBD with overlaps to *rtxB* NBD. All oligonucleotides were purchased from Sigma-Aldrich and diluted in MilliQ water. All PCRs were performed using Q5 DNA polymerase with subsequent DpnI digestion and DNA purification using the Monarch PCR & DNA cleanup kit (all from New England Biolabs). The NucleoSpin plasmid miniprep kit was purchased from Macherey–Nagel and DNA sequencing performed at Microsynth Seqlab.

### Protein expression for secretion experiments

*E. coli* BL21(DE3) chemically competent cells were first transformed^59^ with pSU2726-HlyA and grown on 2xYT agar plates supplemented with 100 µg ml^−1^ ampicillin. A mixture of transformed clones was used to generate a new batch of chemically competent *E. coli* BL21(DE3) cells for a second transformation with one of the pK184 plasmids carrying the genes for *hlyD* and (chimeric) *hlyB* or *rtxB*. Cells were grown on 2xYT agar plates supplemented with 100 µg ml^−1^ ampicillin and 30 µg ml^−1^ kanamycin. Single clones were used to prepare a pre-culture in 5 ml 2xYT medium and cultivated overnight (37 °C and 180 rpm). The pre-culture was used to inoculate 50 ml of 2xYT medium at an OD_600_ of 0.1 in a 300 ml unbaffled shaking flask. Cultures were incubated at 37 °C and 180 rpm to an OD_600_ of 0.8–1.0 and protein expression induced by addition of 1 mM IPTG (isopropyl-β-D-thiogalactopyranoside); additionally, 4 mM CaCl_2_ was added to the media to initiate folding of secreted HlyA. Expression took place for up to 3 h and 1 ml samples were taken and OD_600_ was measured as well. The supernatant was separated from the cells by centrifugation for 2 min at 13,000 xg at room temperature (RT). The cells were resuspended in MilliQ water to normalize the samples in respect of their OD_600_, the same was performed for the supernatants by dilution with MilliQ water. Both cells and supernatant samples were mixed with SDS sample buffer containing 40 mM DTT.

The samples were subjected to an SDS-PAGE with subsequent immunoblot analysis. Before, SDS samples were heated for 5 min to 95 °C. Western blots were performed either in semi-dry (Trans-Blot Turbo, Bio-Rad) or wet blot (Criterion, Bio-Rad). The SDS-PAGE gels were stained using Quick Coomassie Stain solution (Protein Ark). Band intensities of (chimeric) HlyB or RtxB, HlyD and secreted HlyA were quantified from Western blots and Coomassie stained gels using Fiji (version 2.0.0-rc-69/1.52p)^60^. Antibodies used are listed in Supplementary Table S5. Band intensities of HlyA and chHlyB from cells expressing RtxB or chimeric HlyB were normalized to the band intensity of HlyB expressing cells.

### Protein expression and purification of RtxB-NBD

Expression and purification of the NBD from RtxB were performed as described previously for the NBD from HlyB^61^ with slight modifications. In brief, *E. coli* BL21(DE3) was transformed with pPSG122-RtxB-NBD-NHis6 and grown on 2xYT agar plates supplemented with 100 µg ml^−1^ ampicillin. Several clones were added to 50 ml 2xYT medium supplemented with 100 µg ml^−1^ ampicillin for a pre-culture and incubated overnight at 37 °C and 180 rpm. The pre-culture was used to inoculate 2 l of 2xYT supplemented with 100 µg ml^−1^ ampicillin to an OD_600_ of 0.1 in a 5 l baffled shaking flask. Cultures were incubated at 37 °C and 160 rpm to an OD_600_ of 0.6–0.8. The flasks containing the cells were cooled on ice for 20 min. Protein expression was induced by addition of 1 mM L-arabinose and expression took place for 3 h with shaking at 160 rpm and 20 °C.

Cells were harvested by centrifugation at 5,488 xg for 30 min and 4 °C and afterwards resuspended in buffer A1 (25 mM sodium phosphate, 100 mM potassium chloride, 10 mM imidazole, 20% (v/v) glycerol, pH 8). Cells from a total of 6 l expression were used for purification and a cOmplete protease inhibitor cocktail (Sigma-Aldrich) as well as DNase (Sigma-Aldrich) were added. Cells were disrupted by passing them three times through a Microfluidizer M-110P (Microfluidics) at 1.5 kbar. Centrifugation for 90 min at 140,000 xg and 4 °C was used to remove undisrupted cells and cell debris. The supernatant was loaded onto a 5 ml HiTrap Chelating HP column (Cytiva) which was pre-charged with Zn^2+^ and equilibrated with buffer A1. The column was washed with 50 ml buffer A until baseline absorption was reached and the elution was started with a linear gradient over 90 min using buffer A2 (25 mM sodium phosphate, 100 mM potassium chloride, 300 mM imidazole, 20% (v/v) glycerol, pH 8). Fractions containing RtxB-NBD were pooled and concentrated to a final volume of 5 ml using an Amicon Ultra-15 centrifugal filter (MWCO = 10,000 Da, Merck Millipore). The protein solution was centrifuged for 30 min at 17,000 × *g* and 4 °C and subjected to a size exclusion chromatography using a Superdex 200 HiLoad 16/60 prep grade (Cytiva) and buffer B1 (10 mM CAPS-NaOH, 20% (v/v) glycerol, pH 10.4). Again, fractions containing pure RtxB-NBD were pooled and concentrated using an Amicon Ultra-15 centrifugal filter (MWCO = 10,000 Da, Merck Millipore). During this concentration step, the buffer was exchanged step-wise to buffer B2 (100 mM CAPS-NaOH, 20% (v/v) glycerol, pH 10.4). The protein was stored at 4 °C and showed no signs of precipitation even after 4 months.

### ATPase activity assays

ATPase activity was determined using a colorimetric assay as described previously^62^ with minor modifications. In brief, the concentrated and purified RtxB-NBD was diluted in buffer C (100 mM HEPES, 20% (v/v) glycerol, pH 7) directly before starting the reaction to a final concentration of 30 µg ml^−1^. For each reaction, 6 µl of 50 mM MgCl_2_ and 6 µl of ATP solution (final concentrations ranging from 0–8 mM in buffer C) were mixed with 18 µl of RtxB-NBD protein solution (50 µg ml^-1^). For negative controls, buffer C was added instead of MgCl_2_. The reactions were started with the addition of the RtxB-NBD protein solution.

The ATPase reaction mixture was incubated for 90 min at 25 °C and stopped by transferring 25 µl of the reaction mixture to 175 µl of pre-cooled 10 mM H_2_SO_4_ in a 96 well plate. Subsequently, fresh staining solution was prepared (0.096% (w/v) malachite green, 1.48% (w/v) ammonium molybdate, 0.173% (v/v) Tween-20 in 2.36 M H_2_SO_4_) and 50 µl were added. The solution was incubated for 9 min at RT. The amount of free inorganic phosphate was determined by measuring the absorbance at 595 nm using a micro plate reader (iMark Microplate Reader, Bio-Rad). On the same plate, a calibration of the phosphate concentration was performed using Na_2_HPO_4_ with concentrations ranging from 0–500 µM. Data was analyzed using GraphPad Prism 9 Software (GraphPad) and fitted using Eq. (1), the Hill equation:

1 $$v=\frac{{v}_{max}{[S]}^{h}}{{K}_{0.5}^{ h}+{[S]}^{h}}$$

Here, v corresponds to the enzyme velocity, v_max_ is the maximum enzyme velocity, [S] is the substrate concentration, h is the Hill coefficient and K_0.5_ is the substrate concentration, at which half of the enzymes maximum velocity is reached.

### Sequence alignments

Proteins and putative T1SS homologous to the hemolysin system were identified using pBLAST^63^. The sequences that were used as a reference were taken from UniProt with the following UniProt-IDs: HlyB: Q1R2T6, HlyD: Q1R2T7, HlyA: P08715, HlyC: Q1R2T4. The alignments as well as the phylogenetic tree were created with Clustal Omega^38^.

For comparison of the individual ABC transporter domains from HlyB and RtxB, pairwise protein sequence alignments were performed using the GGSEARCH2SEQ tool^38^. The sequence for RtxB was taken from UniProt (UniProt-ID: F5S9L7).

### Structure predictions of RtxA and HlyA using AlphaFold2

Secondary structure prediction of the secretion signal sequences of HlyA and RtxA were performed using the Quick2D tool^64^. The helical wheel projections were performed with NetWheel^65^.

The structure of HlyA from *E. coli* (UniProt-ID: P08715) as well as HlyB and the chimeric transporters were predicted using AlphaFold2^39^ with standard settings. The structure of RtxA is accessible via the AlphaFold identifier AF-A1YKW7-F1.

### Supplementary Information

Supplementary Information.

## Data Availability

We uploaded the SAXS data to the Small Angle Scattering Biological Data Bank (SASBDB)^66^, with the accession code **SASDS56**.
