# Supplementary material for: Type 1 secretion necessitates a tight interplay between all domains of the ABC transporter
Source: Sci Rep. 2024 Apr 18;14:8994. doi: 10.1038/s41598-024-59759-0 (PMC11026475; doi:10.1038/s41598-024-59759-0)
Supplement: Supplementary file 1 — Supplementary Information. [file 41598_2024_59759_MOESM1_ESM.pdf]

SUPPLEMENTARY INFORMATION

Type 1 secretion necessitates a tight interplay between all domains of the ABC transporter

Manuel T. Anlauf[1], Florestan L. Bilsing[1], Jens Reiners[2], Olivia Spitz[1†], Eymen Hachani[1] , Sander H.J. Smits[1,2] and Lutz Schmitt[1*]

[1]Institute of Biochemistry, Heinrich Heine University Düsseldorf, Universitätsstraße 1, 40225 Düsseldorf, Germany

[2]Center for Structural Studies, Heinrich Heine University Düsseldorf, Universitätsstraße 1, 40225 Düsseldorf, Germany

[†]present adress: INCONSULT, Duisburg, Germany

*Corresponding author: Lutz Schmitt, Institute of Biochemistry, Heinrich Heine University Düsseldorf, Universitätsstraße 1, 40225 Düsseldorf, Germany, Tel.: +49 211 81-10773, E-Mail: lutz.schmitt@hhu.de

Supplemental Material and Methods

Small Angle X-ray Scattering (SAXS)

The SAXS data was collected on the P12 beamline (PETRA III, DESY Hamburg[1]). The sample to detector distance of the P12 beamline was 3.00 m, resulting in an achievable q-range of 0.03 – 4.5 nm^{-1}. The measurements were performed at 10°C with a protein concentration of 1.93 mg ml^{-1} of RtxB-NBD. 40 frames were collected with an exposer time of 0.095 sec $frame^{-1}$. Data were collected on relative scale.

All used programs for data processing were part of the ATSAS Software package (Version 3.0.5)[2]. Primary data reduction was performed with the program PRIMUS[3]. The forward scattering *I(0)* and the radius of gyration (R_g) were determined with the Guinier approximation[4]. The program GNOM[5] was used to estimate the maximum particle dimension (D_{max}) with the pair-distribution function *p(r)*. Low resolution *ab initio* models were calculated with GASBOR[6]. Superimposing of the RtxB-NBD AlphaFold2[7,8] model was done with the program SUPCOMB[9].

Table S1: Identity and characteristics of putative T1SS components from 25 different organisms. Proteins were identified by pBLAST search. The Number (No.) of GG repeats is displayed in form of a range and depends on how strict the RTX motif GGxGxDxUx (U being a large hydrophobic residue and x being any amino acid) is applied[10]. If multiple identities and sizes for the HlyA homolog are displayed, more than one RTX protein was identified.

	Identity compared to [%]			Putative RTX protein	
**Organism**	**HlyB**	**HlyD**	**HlyA**	**Size [aa]**	**No. of GG repeats**
Xylella fastidiosa	61	41	37	1,814	16-18
Xanthomonas axonopodis	63	38	38	2,512	36-38
Lysobacter antibioticus	64	37	42	574	9-11
Aeromonas diversa CDC 2478-85	65	38	43	351	9-10
Gallibacterium anatis	70	49	29	2,038	5-6
Avibacterium paragallinarum	69	48	32	2,286	15
Cronobacter malonaticus	69	46	36	866	6
Aggregatibacter actinomycetemcomitans	84	68	50	1,051	7
Mannheimia haemolytica	82	61	43	953	5
Bibersteinia trehalosi	82	59	42	955	6
Pasteurella aerogenes	83	62	52	1,049	6-9
Actinobacillus equuli subsp. Haemolyticus	86	64	47	987	4
Morganella morganii	90	81	80	1,024	5-6
Proteus vulgaris	92	95	46	598	6
Vibrio parahaemolyticus	92	81	82	986	8-9
Enterobacter cloacae	98	98	97	1,024	6-7
Serratia sp. Leaf51	69	42	33; 34	965; 2,893	9; 12-14
Cardiobacterium valvarum	70	41	34; 35; 41; 49	217; 569; 665; 558	4; 8-9; 2; 5-6
Vitreoscilla sp. SN6	70	42	44	444	10
Acinetobacter baumannii	69	44	52	3,298	49-58
Moraxella bovis	69	41	43	927	5
Kingella kingae	**71**	**40**	**42**	**956**	**6**
Alysiella crassa	70	41	31	248	4
Snodgrassella alvi	71	45	50	895	24
Neisseria sp. oral taxon 020	70	43	38; 46; 49	1,605; 636; 188	14; 3-5; 4-5

Table S2: Overall SAXS Data for RtxB-NBD.

**SAXS Device**	**P12, PETRA III, DESY Hamburg[1]**
**Data collection parameters**	
Detector	PILATUS 6 M (423.6 x 434.6 mm^2)
Detector distance (m)	3.0
Beam size	120 µm x 200 µm
Wavelength (nm)	0.124
Sample environment	Quartz glass capillary, 1 mm ø
s range (nm^{-1})‡	0.03 – 4.5
**Sample**	**RtxB-NBD**
Organism	*Kingella kingae*
UniProt ID	F5S9L7
Mode of measurement	batch
Temperature (°C)	10
Exposure time per frame (s)	0.095 (40 Frames)
Protein buffer	100 mM HEPES pH 8.0, 10 % (v/v) Glycerol
Protein concentration (mg/ml)	1.93
**Structural parameters**	
I(0) from P(r)	0.02
R_g (real-space from P(r)) (nm)	2.20
s-range for GNOM fit (nm^{-1})	0.133 – 4.181
I(0) from Guinier fit	0.02
s-range for Guinier fit (nm^{-1})	0.133 – 0.593
R_g (from Guinier fit) (nm)	2.19
points from Guinier fit	1 - 160
D_{max} (nm)	7.60
POROD volume estimate (nm^3)	47.55
**Molecular mass (kDa)**	
From *I*(0)	25.77
From Qp[11]	20.36
From MoW2[12]	21.26
From Vc[13]	24.92
Bayesian Inference[14]	23.05
From GNNOM[15]	30.70
From POROD	29.72
From sequence	27.68 (monomer)<br>55.36 (dimer)
**Structure Evaluation**	
GASBOR fit χ^2	1.082
CRYSOLfit χ^2	1.445
Ambimeter score	1.362
**Software**	
ATSAS Software Version[2]	3.0.5
Primary data reduction	PRIMUS[3]
Data processing	GNOM[5]
Ab initio modelling	GASBOR[6]
Superimposing	SUPCOMB[9]
Structure evaluation	AMBIMETER[16] / CRYSOL[17]
Model visualization	PyMOL[18]

‡s = 4πsin(θ)/λ, 2θ – scattering angle, n.d. not determined

Table S3: Plasmids used in this study.

Plasmid name	Backbone	Encoded genes	Source
pK184-HlyBD	pK184	*hlyB*, *hlyD*	[19]
pK184-HlyBD-KEE	pK184	*hlyB-KEE, hlyD*	This study
pK184-HlyBD-EKE	pK184	*hlyB-EKE, hlyD*	This study
pK184-HlyBD-EEK	pK184	*hlyB-EEK, hlyD*	This study
pK184-HlyBD-KKE	pK184	*hlyB-KKE, hlyD*	This study
pK184-HlyBD-KEK	pK184	*hlyB-KEK, hlyD*	This study
pK184-HlyBD-EKK	pK184	*hlyB-EEK, hlyD*	This study
pK184-RtxB-HlyD	pK184	*rtxB, hlyD*	This study
pSU2726-HlyA	pSU2726	*hlyA*	[20]
pPSG122-RtxB-NBD-NHis6	pBAD18	*rtxB-NBD*	This study

Table S4: Oligonucleotides used in this study. Overhangs for Gibson assembly are underlined.

Name	Details	Sequence (5'→3')	Plasmid
lin-pK-fw	Linearization of the pK184-HlyBD plasmid without *hlyB*	CATGACTGTTTCCTGTGTGAAATTG	pK184-RtxB-HlyD
lin-pK-rev		TAACAGAAAGAACAGAAGAATATGAAAAC	
pK-Ins-RtxB-fw	Amplification of *rtxB* with overhangs to pK184	CAGGAAACAGTCATGGATAAAACTTCTCAACCCGC	
pK-Ins-RtxB-rev		CTTCTGTTCTTTCTGTTACCCATTCTGTAAATCATACAAATAACG	
RtxB-CLD-fw	Amplification of *rtxB* CLD	GATAAAACCTCTCAACCCGC	pK184-HlyBD-KEE
RtxB-CLD-rev		GACAAAAATCATTTTGCCTGAATATC	
pK-RtxB-CLD-Ins-fw	Amplification of pK184-HlyBD without *hlyB* CLD with overhangs to *rtxB* CLD	GCAAAATGATTTTTGTCGCTTCCCGTTCTTCTGTTG	
pK-RtxB-CLD-Ins-rev		GTTGAGAGGTTTTATCCATGACTGTTTCCTGTGTG	
RtxB-TMD-fw	Amplification of *rtxB* TMD	TTAGAAGTGCTGCTGGTGTC	pK184-HlyBD-EKE
RtxB-TMD-rev		TAACTGTGCCAAACGAATCAC	
pK-RtxB-TMD-Ins-fw	Amplification of pK184-HlyBD without *hlyB* TMD with overhangs to *rtxB* TMD	GTTTGGCACAGTTATGGCAGGATTTCCAGCAG	
pK-RtxB-TMD-Ins-rev		AGCAGCACTTCTAAAAATATTCTCCTGTATTTTATAATGGCAG	
RtxB-NBD-fw	Amplification of *rtxB* NBD	ATTACTTTTGAACACGTTGATTTTAG	pK184-HlyBD-EEK
RtxB-NBD-rev		CCCATTCTGTAAATCATACAAATAAC	
pK-RtxB-NBD-Ins-fw	Amplification of pK184-HlyBD without *hlyB* NBD with overhangs to *rtxB* NBD	TGATTTACAGAATGGGTAACAGAAAGAACAGAAGAATATG	
pK-RtxB-NBD-Ins-rev		CGTGTTCAAAAGTAATATCACCATTAATTTCCGG	
HlyB-CLD-fw	Amplification of *hlyB* CLD	GCGAATTCTGATTCTTGTCATAAAATTG	pK184-HlyBD-EKK
HlyB-CLD-rev		GATAAGAATAATATGCCCCTGATATAACG	
pK-HlyB-CLD-Ins-fw	Amplification of pK184-RtxB-HlyD without *rtxB* CLD with overhangs to *hlyB* CLD	GGGCATATTATTCTTATCGCATCTCGCGCATCCGTG	
pK-HlyB-CLD-Ins-rev		AAGAATCAGAATTCGCCATGACTGTTTCCTGTGTGAAATTGTTATCC	
HlyB-TMD-fw	Amplification of *hlyB* TMD	ATTGAAACCCTTGTTGTGTCTG	pK184-HlyBD-KEK
HlyB-TMD-rev		GATTTGTGCAAGGCGAATAAC	
pK-HlyB-TMD-Ins-fw	Amplification of pK184-RtxB-HlyD without *rtxB* TMD with overhangs to *hlyB* TMD	GCCTTGCACAAATCTGGCAGGATTTTCAGCAAG	
pK-HlyB-TMD-Ins-rev		CAACAAGGGTTTCAATAAAAATGCGGCGATATTTAATCAC	
HlyB-NBD-fw	Amplification of *hlyB* NBD	ATCACTTTTCGTAATATCCGGTTTC	pK184-HlyBD-KKE
HlyB-NBD-rev		GTCTGACTGTAACTGATATAAGTAAC	
pK-HlyB-NBD-Ins-fw	Amplification of pK184-RtxB-HlyD without *rtxB* NBD with overhangs to *hlyB* NBD	CAGTTACAGTCAGACTAACAGAAAGAACAGAAGAATATGAAAAC	
pK-HlyB-NBD-Ins-rev		GATATTACGAAAAGTGATGTCGCCTTGAATATCGGG	
pPSG-RtxB-NBD-Ins-fw	Amplification of pPSG122-HlyB-NBD-NHis6 without *hlyB* NBD with overhangs to *rtxB* NBD	TGATTTACAGAATGGGTAAGAATTCGAGCTCGGTAC	pPSG122-RtxB-NBD-NHis6
pPSG-RtxB-NBD-Ins-rev		ACGTGTTCAAAAGTAATATCGTGATGGTGATGGTG	

Table S5: List of used antibodies.

Name	Description	Host	Dilution
Anti-HlyA	Polyclonal, targets the N-terminal secretion signal of pro-HlyA, purified	Rabbit	1 : 1,000
Anti-HlyB	Polyclonal, targets the NBD of HlyB, purified	Rabbit	1 : 4,000
Anti-HlyD	Polyclonal, targets the periplasmic part of HlyD, serum	Rabbit	1 : 4,000
Anti-Rabbit-HRP	Polyclonal, targets rabbit IgG, HRP-conjugated, purified (Thermo Scientific)	Goat	1 : 20,000

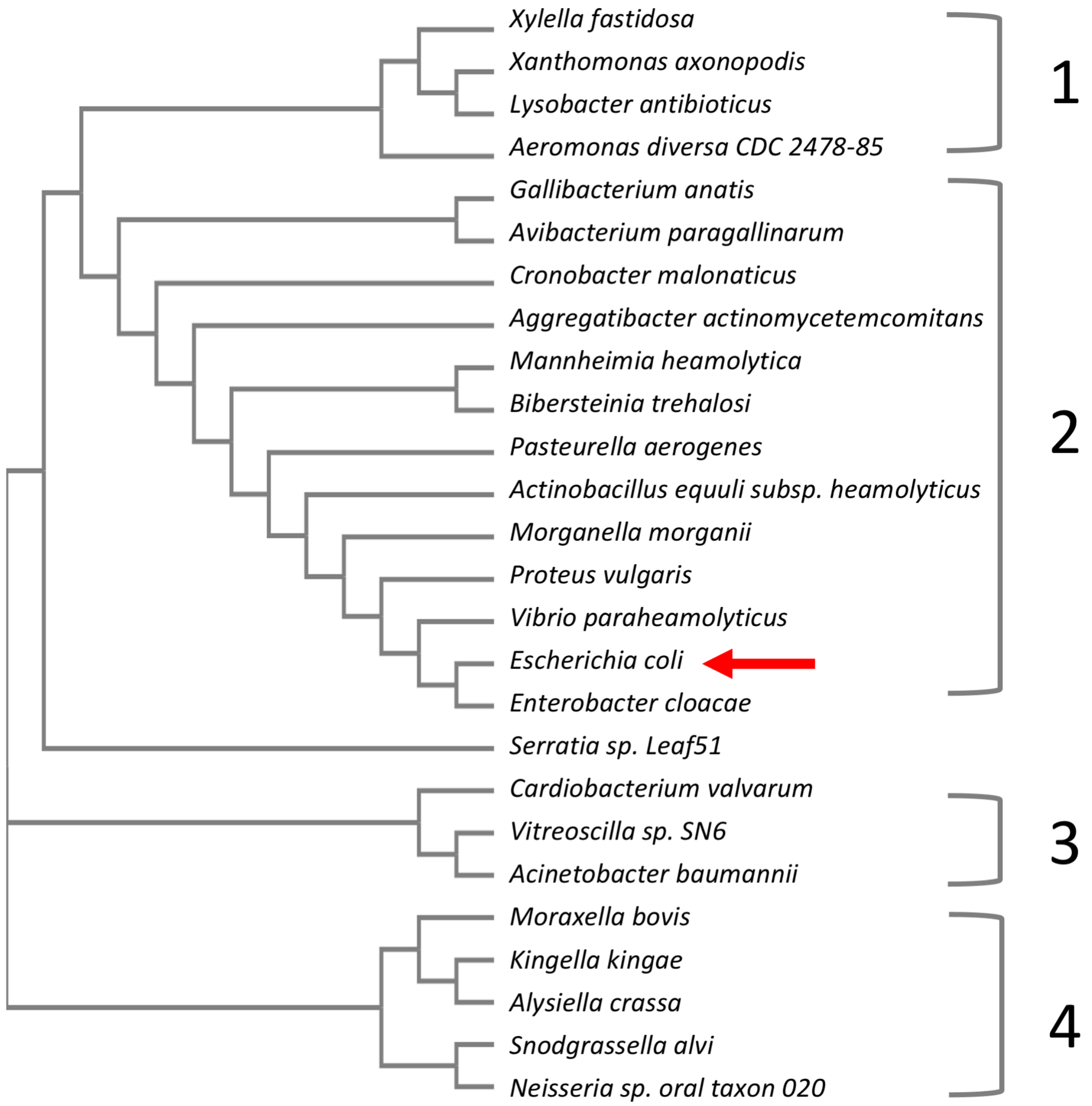

Figure S1: Phylogenetic tree of organisms containing transporters homologous to HlyB. The primary sequence of T1SS transporters from the depicted organisms was used in an alignment performed with Clustal Omega[21]. The organisms can be divided into four groups based on their relation. *Escherichia coli* is marked with a red arrow. Note that the length of the branches does not relate to the degree of relation.

a

```
HlyA           974 — NPLINEISKIISAAGSF - 990

SS_PSIPRED             HHHHHHHHHHHH
SS_PSSPRED4            HHHHHHHHHHHH
SS_DEEPCNF            HHHHHHHHHHHHH
SS_NETSURFP2          HHHHHHHHHHHHH
```

b

```
RtxA           913 — GNLASTLNKLIESMASF - 929

SS_PSIPRED             HHHHHHHHHHHHH
SS_PSSPRED4            HHHHHHHHHHHHHH
SS_DEEPCNF             HHHHHHHHHHHHH
SS_NETSURFP2           HHHHHHHHHHHHH
```

Figure S2: Prediction of secondary structures in the secretion signal sequences of HlyA (**a**) and RtxA (**b**) using the Quick2D tool. SS_PSIPRED[22], SS_PSSPRED4[23], SS_DEEPCNF[24] and SS_NETSURFP2[25] are different prediction algorithms. 'H' indicates amino acids, which are predicted to form an α-helix.

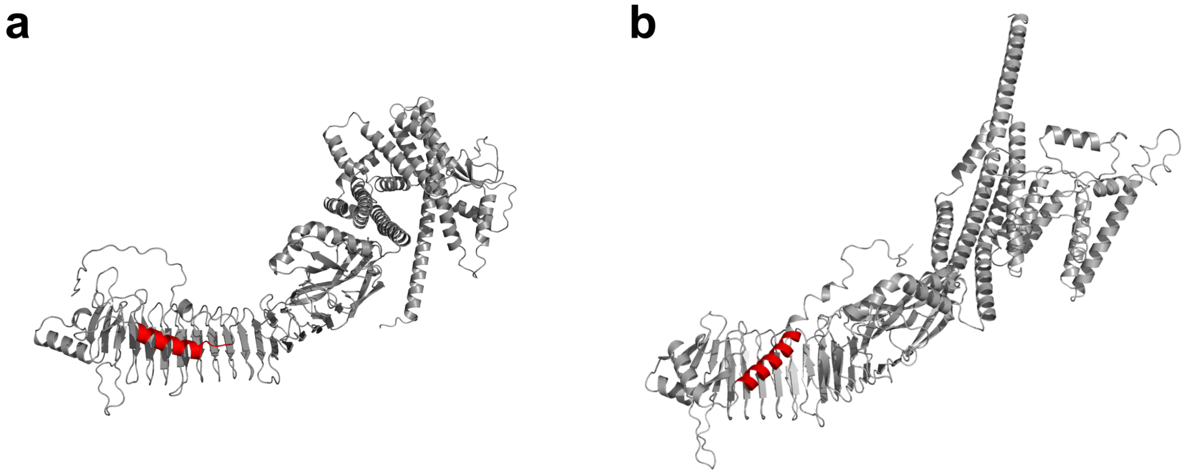

Figure S3: Models of pro-HlyA (**a**) and pro-RtxA (**b**) as predicted by AlphaFold2[8]. The C-terminal amphipathic helix of the secretion signal sequence of both toxins is shown in red.

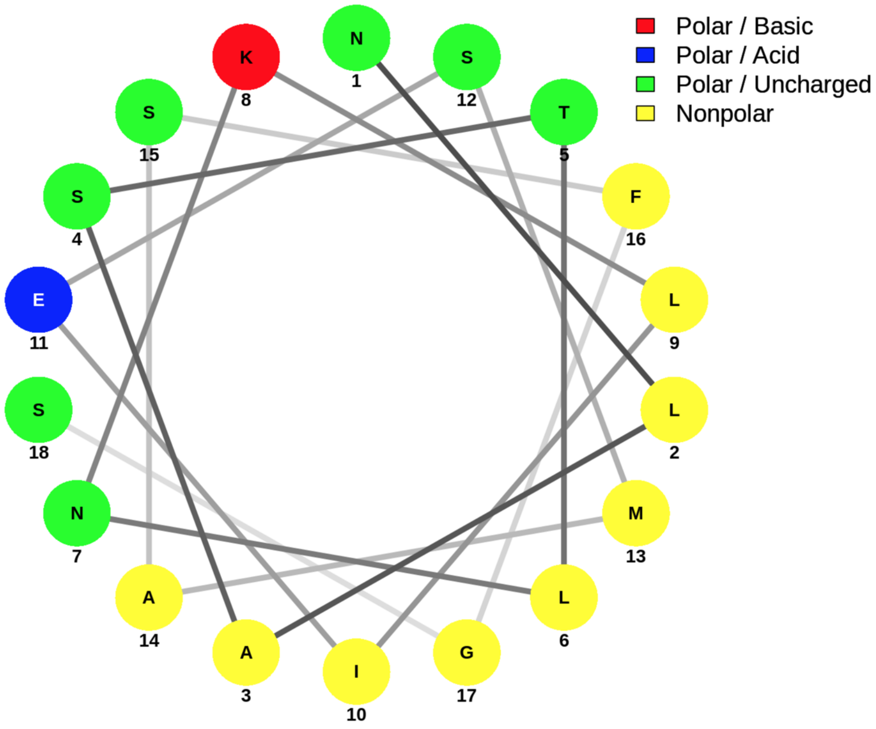

Figure S4: Helical wheel projection of the putative amphipathic helix in the secretion signal of RtxA. The image was created using NetWheel[26]. Nonpolar residues are shown in yellow, polar residues in green, acidic residues in blue and basic residues in red.

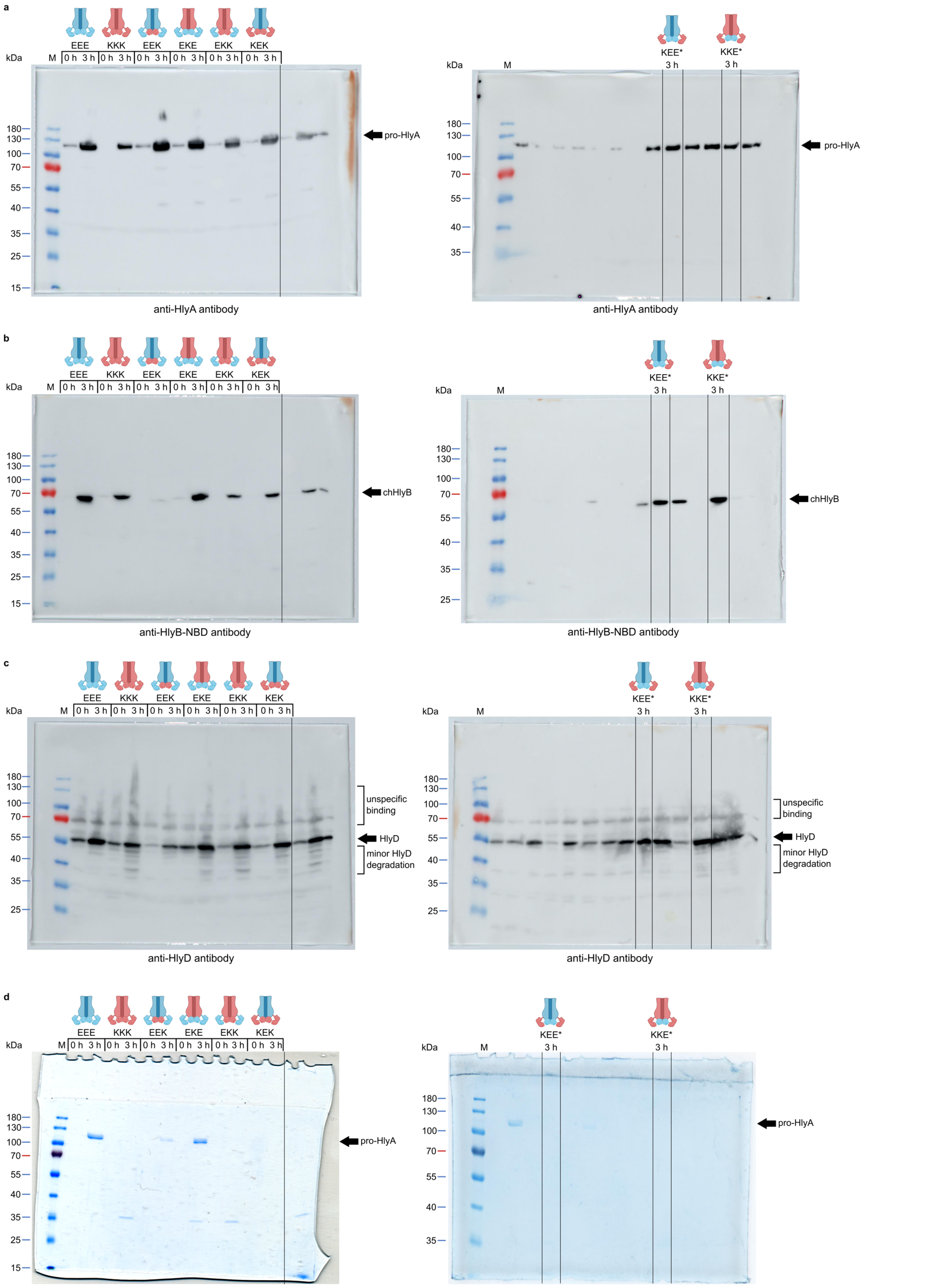

Figure S5: Uncropped Western Blots and SDS-PAGE gels shown in Figure 1. Vertical crop sides are indicated as black lines for better distinction between lanes of samples shown in the main text and lanes containing unrelated samples.

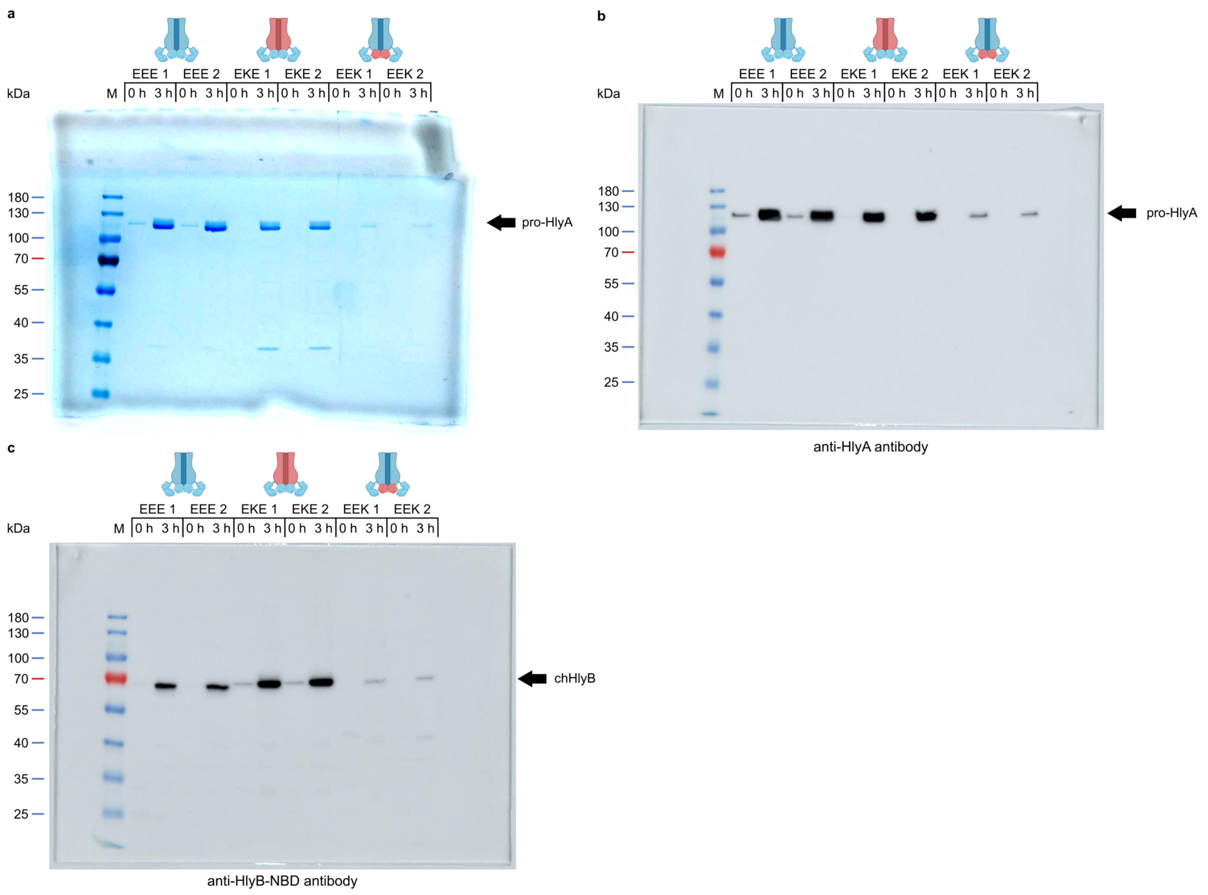

Figure S6: Uncropped Western Blots and SDS-PAGE gels shown in Figure 3.

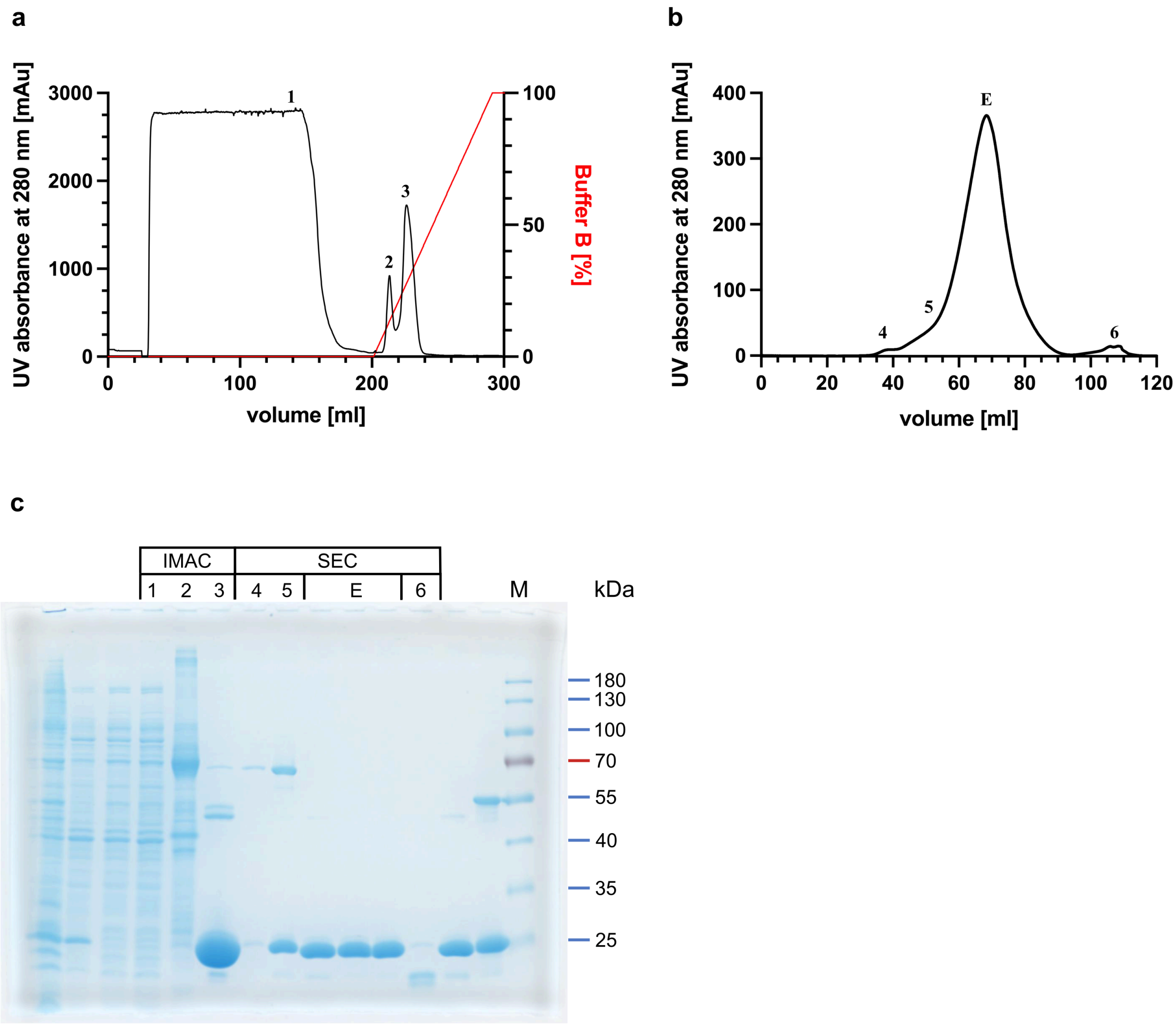

Figure S7: Purification of RtxB-NBD. The N-terminally fused 6xHis-tag allowed an immobilized metal affinity chromatography (IMAC, **a**); 1: Flow through; 2: Wash; 3: Elution. After washing to baseline, a gradient elution was performed (red, 100% Buffer = 300 mM imidazole). The elution peak was concentrated and applied to a size exclusion chromatography (SEC, **b**); 4,5,6: Contamination; E: Elution of target protein. (**c**): SDS-PAGE analysis of protein samples from IMAC and SEC with Coomassie staining. RtxB-NBD-NHis6 has a size of 27,676 Da. M: Protein marker, the approx. size of the marker proteins is given on the right.

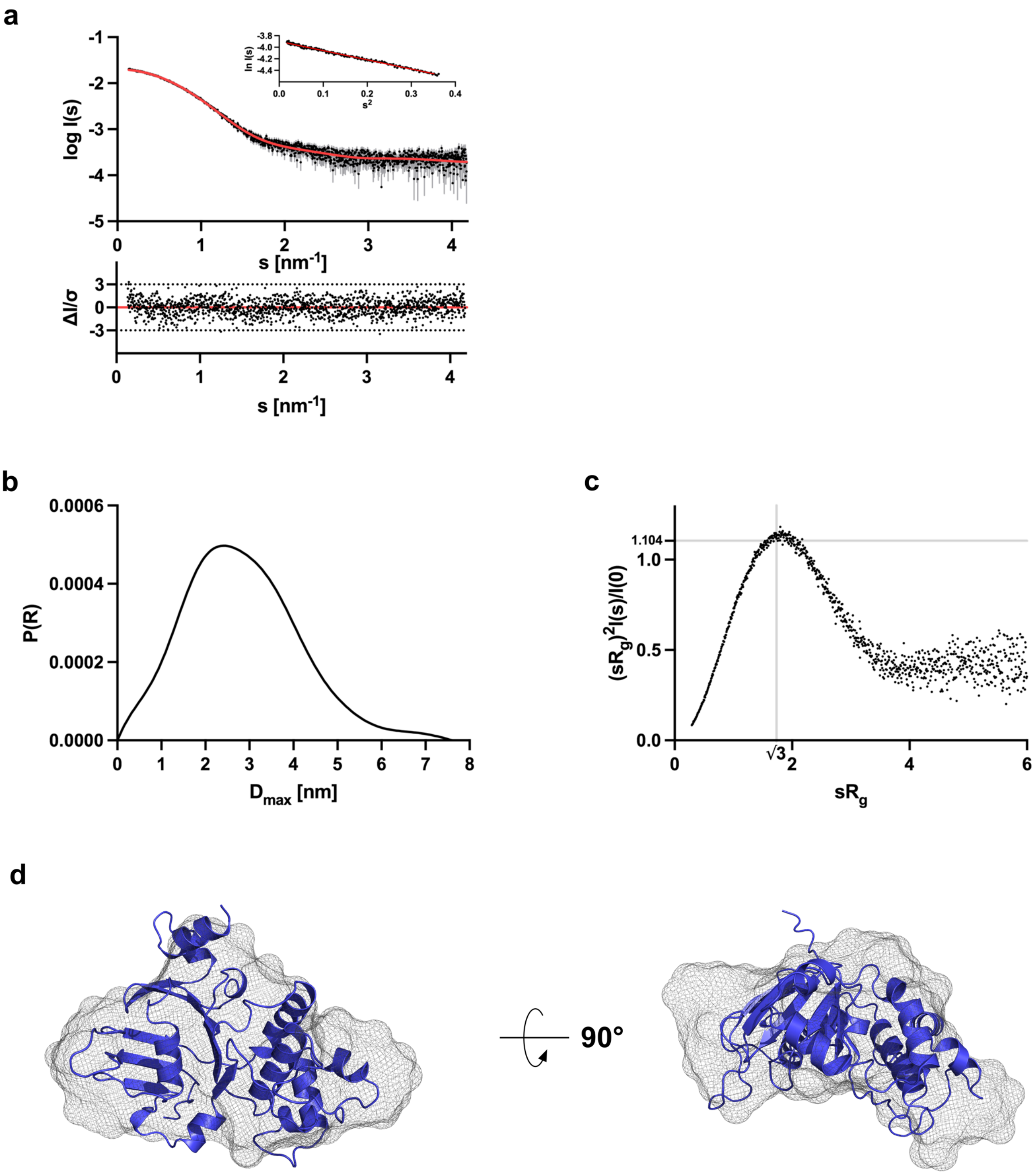

Figure S8: Small-angle X-ray scattering data from RtxB-NBD. (**a**): Scattering data of RtxB-NBD. Experimental data is shown in black dots, with grey error bars. The GASBOR *ab initio* model fit is shown as a red line, below is the residual plot of the data. The Guinier plot is added in the upper right corner. (**b**): Pair distance distribution function *p(r)* revealed RtxB-NBD to be a folded, monomeric, almost globular particle in solution. (**c**): Dimensionless Kratky plot of RtxB-NBD showed a compact particle, as globular particles exhibit a peak at √3 with a maximum at 1.104 (marked with grey lines). (**d**): The AlphaFold2 model is shown in blue, and the GASBOR *ab-initio* envelope is shown as a grey mesh (GASBOR fit χ^2 = 1.082). The unstructured N-terminus sticking out of the mesh contains the 6xHis-tag.

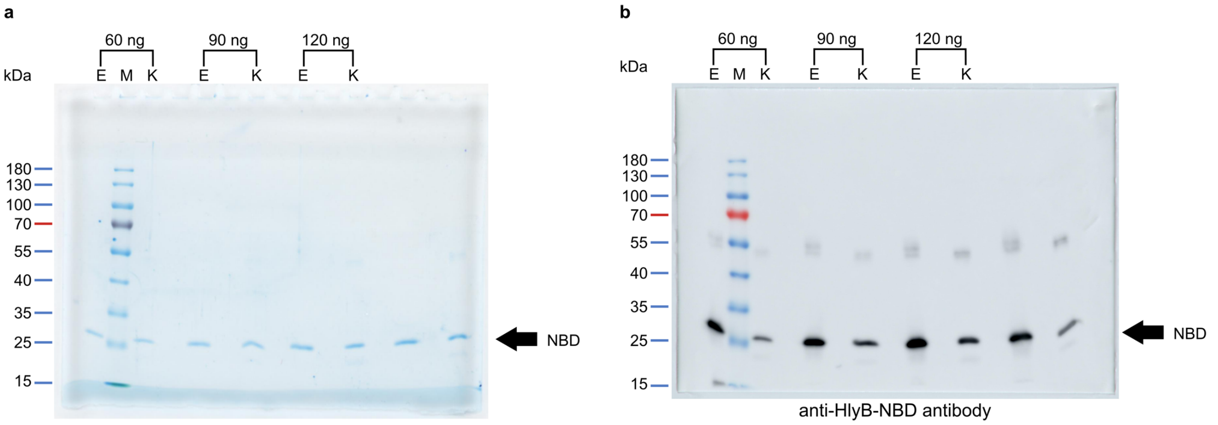

Figure S9: Comparative quantification of the NBDs from HlyB and RtxB. Equal amounts of purified protein ranging from 60-120 ng were analyzed via SDS-PAGE (**a**) and immunoblot using the HlyB-NBD antibody (**b**). The signal intensity of the HlyB-NBD antibody for RtxB-NBD was divided by the HlyB-NBD signal intensity and resulted in a factor of 0.36 ± 0.10. M: Protein marker, the approx. size of the marker proteins is given on the left; E: HlyB-NBD; K: RtxB-NBD.

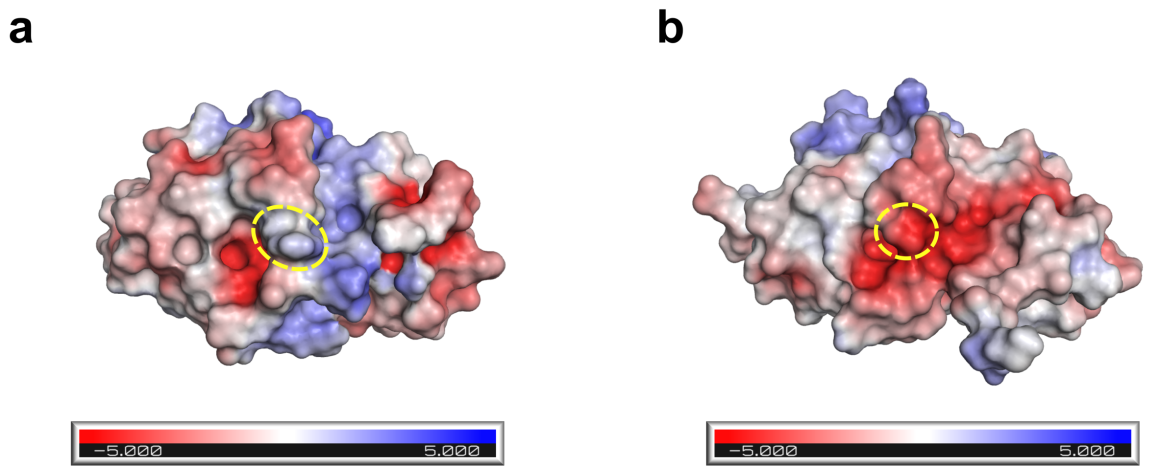

Figure S10: Surface charge of CLDs from chimeric transporters. Depicted are the CLD from HlyB (**a**) and from RtxB (**b**). Positive surface charge is shown blue, while negative charge is shown in red. The yellow circle indicates the position of R82 in case of HlyB and of D81 in case of RtxB. Surface charge was calculated in PyMOL using the APBS tool.

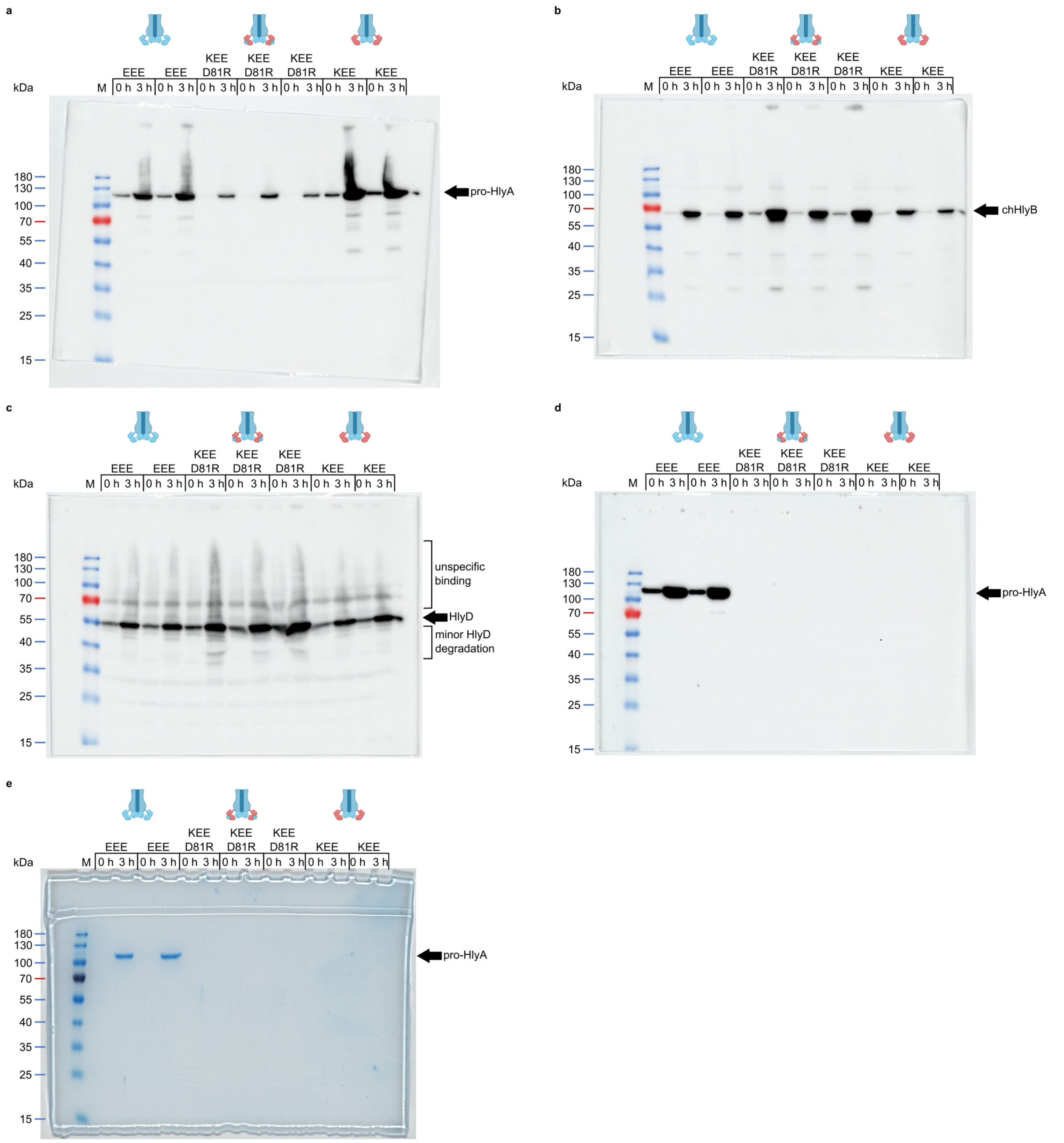

Figure S11: Secretion of pro-HlyA by HlyB-KEE-D81R. Immunoblot analysis of whole *E. coli* cells expressing the indicated chHlyB show the presence of pro-HlyA (**a**), chHlyB (**b**) and HlyD (**c**). Immunoblot (**d**) and SDS-PAGE analysis (**e**) of pro-HlyA from supernatants of the aforementioned cells. Cell and supernatant samples were diluted to match the same OD_{600}. Schematic representation of the chHlyB variants are depicted above the respective chimera, the point mutation is indicated with a star. Blue domains originate from HlyB (*E. coli*) and red domains from RtxB (*K. kingae*). Signals corresponding to unspecific binding and degradation are marked in case of the anti-HlyD western blot. The point mutation D81R introduced into the CLD of HlyB-KEE does not restore the ability to secrete pro-HlyA. M: Protein marker, the approximated molecular weight of the marker proteins is given on the left; x h: time after induction, when the samples were taken.

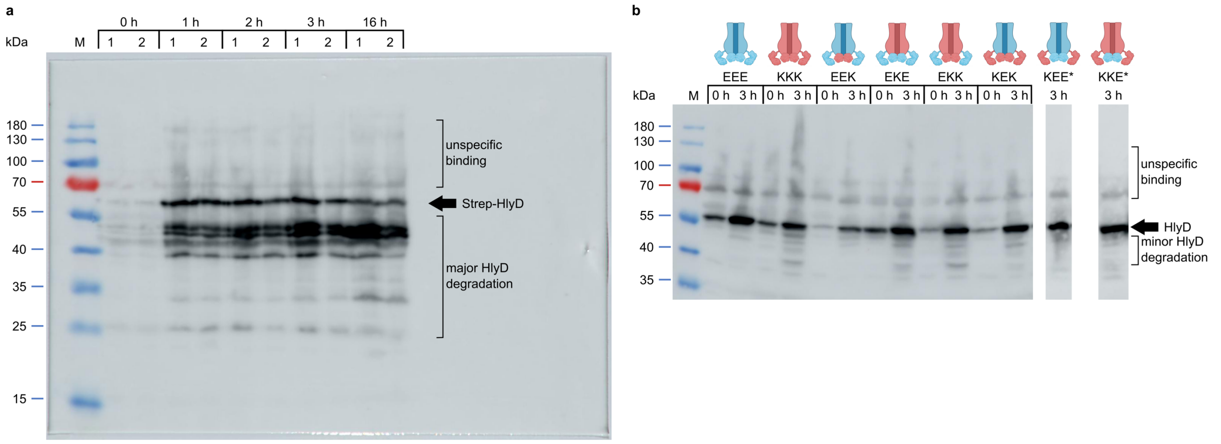

Figure S12: Degradation of HlyD. (**a**) Expression of Strep-tagged HlyD without the presence of HlyB in two replicates (1 & 2). Samples were taken before induction (0 h) as well as after 1 h, 2 h, 3 h and 16 h of expression. HlyD severely degrades when HlyB is not present and no secretion complex can be formed. (**b**) Figure 1 C from the main text is shown here for easier comparison.

Supplemental References

1. Blanchet, C. E. *et al.* Versatile sample environments and automation for biological solution X-ray scattering experiments at the P12 beamline (PETRA III, DESY). *J. Appl. Crystallogr.* **48**, 431-443 (2015).
2. Manalastas-Cantos, K. *et al.* ATSAS 3.0: expanded functionality and new tools for small-angle scattering data analysis. *J. Appl. Crystallogr.* **54**, 343-355 (2021).
3. Konarev, P. V., Volkov, V. V., Sokolova, A. V., Koch, M. H. J. & Svergun, D. I. PRIMUS: a Windows PC-based system for small-angle scattering data analysis. *J. Appl. Crystallogr.* **36**, 1277-1282 (2003).
4. Guinier, A. Small-angle X-ray diffraction: application to the study of ultramicroscopic phenomena. *Ann. Phys.* **11**, 161-237 (1939).
5. Svergun, D. I. Determination of the regularization parameter in indirect-transform methods using perceptual criteria. *J. Appl. Crystallogr.* **25**, 495-503 (1992).
6. Svergun, D. I., Petoukhov, M. V. & Koch, M. H. Determination of domain structure of proteins from X-ray solution scattering. *Biophys. J.* **80**, 2946-2953 (2001).
7. Mirdita, M. *et al.* ColabFold: making protein folding accessible to all. *Nat. Methods* **19**, 679-682 (2022).
8. Jumper, J. *et al.* Highly accurate protein structure prediction with AlphaFold. *Nature* **596**, 583-589 (2021).
9. Kozin, M. B. & Svergun, D. I. Automated matching of high- and low-resolution structural models. *J. Appl. Crystallogr.* **34**, 33-41 (2001).
10. Linhartová, I. *et al.* RTX proteins: a highly diverse family secreted by a common mechanism. *FEMS Microbiol. Rev.* **34**, 1076-1112 (2010).
11. Porod, G. Die Röntgenkleinwinkelstreuung von dichtgepackten kolloiden Systemen - 1 Teil. *Kolloid Z.* **124**, 83-114 (1951).
12. Fischer, H., Neto, M. D., Napolitano, H. B., Polikarpov, I. & Craievich, A. F. Determination of the molecular weight of proteins in solution from a single small-angle X-ray scattering measurement on a relative scale. *J. Appl. Crystallogr.* **43**, 101-109 (2010).
13. Rambo, R. P. & Tainer, J. A. Accurate assessment of mass, models and resolution by small-angle scattering. *Nature* **496**, 477-481 (2013).
14. Hajizadeh, N. R., Franke, D., Jeffries, C. M. & Svergun, D. I. Consensus Bayesian assessment of protein molecular mass from solution X-ray scattering data. *Sci. Rep.* **8**, 7204 (2018).
15. Molodenskiy, D. S., Svergun, D. I. & Kikhney, A. G. Artificial neural networks for solution scattering data analysis. *Structure* **30**, 900-908 (2022).
16. Petoukhov, M. V. & Svergun, D. I. Ambiguity assessment of small-angle scattering curves from monodisperse systems. *Acta Crystallogr. D Biol. Crystallogr.* **71**, 1051-1058 (2015).
17. Svergun, D., Barberato, C. & Koch, M. H. J. CRYSOL - A program to evaluate X-ray solution scattering of biological macromolecules from atomic coordinates. *J. Appl. Crystallogr.* **28**, 768-773 (1995).
18. Schrödinger, L. The PyMOL molecular graphics system, version 2.5. (2022).
19. Bakkes, P. J., Jenewein, S., Smits, S. H., Holland, I. B. & Schmitt, L. The rate of folding dictates substrate secretion by the *Escherichia coli* hemolysin type 1 secretion system. *J. Biol. Chem.* **285**, 40573-40580 (2010).

20. Thomas, S., Smits, S. H. & Schmitt, L. A simple in vitro acylation assay based on optimized HlyA and HlyC purification. *Anal. Biochem.* **464**, 17-23 (2014).
21. Madeira, F. *et al.* Search and sequence analysis tools services from EMBL-EBI in 2022. *Nucleic Acids Res.* **50**, W276-W279 (2022).
22. Jones, D. T. Protein secondary structure prediction based on position-specific scoring matrices. *J. Mol. Biol.* **292**, 195-202 (1999).
23. Yan, R., Xu, D., Yang, J., Walker, S. & Zhang, Y. A comparative assessment and analysis of 20 representative sequence alignment methods for protein structure prediction. *Sci. Rep.* **3**, 2619 (2013).
24. Wang, S., Peng, J., Ma, J. & Xu, J. Protein secondary structure prediction using deep convolutional neural fields. *Sci. Rep.* **6**, 18962 (2016).
25. Klausen, M. S. *et al.* NetSurfP-2.0: Improved prediction of protein structural features by integrated deep learning. *Proteins: Struct. Funct. Bioinform.* **87**, 520-527 (2019).
26. Mól, A. R., Castro, M. S. & Fontes, W. NetWheels: A web application to create high quality peptide helical wheel and net projections. *BioRxiv*, 416347 (2018).
27. Zhao, H., Lee, J. & Chen, J. The hemolysin A secretion system is a multi-engine pump containing three ABC transporters. Cell 185, 3329-3340 (2022).
28. Kieuvongngam, V. *et al.* Structural basis of substrate recognition by a polypeptide processing and secretion transporter. *eLife* **9** (2020).
